# Supplementary material for: Age-dependent topic modeling of comorbidities in UK Biobank identifies disease subtypes with differential genetic risk
Source: Nat Genet. 2023 Oct 9;55(11):1854–65. doi: 10.1038/s41588-023-01522-8 (PMC10632146; doi:10.1038/s41588-023-01522-8)
Supplement: Supplementary file 1 — Supplementary Note, Supplementary Tables 1–21 and Supplementary Figs. 1–26. [file 41588_2023_1522_MOESM1_ESM.pdf]

nature genetics

Article https://doi.org/10.1038/s41588-023-01522-8

Age-dependent topic modeling of comorbidities in UK Biobank identifies disease subtypes with differential genetic risk

In the format provided by the authors and unedited

Supplementary Note

1 Secondary analyses

Simulation:

We simulated 61,000 disease diagnoses spanning 20 diseases in 10,000 individuals, using the ATM generative model; we aimed to choose simulation parameters that resemble real data. In detail, the average number of disease diagnoses per individual (6.1), ratio of #individuals/#diseases (500), topic loadings, and standard deviation in age at diagnosis (8.5 years for each disease) were chosen to match empirical UK Biobank data; we varied the number of topics, number of individuals, and number of diseases in secondary analyses (see below). We note that AUPRC is larger when classifying the minority subtype; results using the second subtype as the classification target are also provided (Supplementary Fig. 2).

We performed three additional secondary analyses. First, we varied the number of individuals, number of diseases, or number of disease diagnoses per individual. ATM continued to outperform LDA in each case, although increasing the number of individuals or the number of disease diagnoses per individual did not always increase AUPRC (Supplementary Fig. 4B). Second, we performed simulations in which we increased the number of subtypes from two to five and changed the number of diseases to 50, and compared ATM models trained using different numbers of topics (in 80% training data) by computing the prediction odds ratio; we used the prediction odds ratio (instead of AUPRC) in this analysis both because it is a better metric to evaluate the overall model fit to the data, and because it is unclear how to compare AUPRC across scenarios of varying topic numbers (see Supplementary Table 1). We confirmed that the prediction odds ratio was maximised using five topics, validating the use of the prediction odds ratio for model selection (Supplementary Fig. 5A). Third, we computed the accuracy of inferred topic loadings, topic weights, and grouping accuracy (defined as proportion of pairs of diseases truly belonging to the same topic that ATM correctly assigned to the same topic), varying the number of individuals and number of diseases diagnoses per individual. We determined that ATM also performed well under these metrics (Supplementary Fig. 5B-E).

Age-dependent comorbidity profiles in the UK Biobank

We performed three additional secondary analyses to validate the integrity and reproducibility of inferred comorbidity topics. First, we reached similar conclusions on model selection using evidence lower bounds[1] (ELBO; see Supplementary Table 1) as prediction odds ratios; ATM with 10 topics fits the data optimally (Supplementary Fig. 6. Second, we confirmed that collapsed variational inference [2] outperformed mean-field variational inference [3] (Supplementary Fig. 7). Third, we computed a co-occurrence odds ratio evaluating whether diseases grouped into the same topic by ATM in the training data have higher than random probability of co-occurring in the testing data (Supplementary Table 1). The co-occurrence odds ratio is consistently above one and increases with the number of comorbid diseases, for each inferred topic (Supplementary Fig. 8).

Age-dependent comorbidity profiles in All of Us

We note 3 key differences between All of Us and UK Biobank data: (i) All of Us contains primary care and hospital data encoded using SNOMED clinical terms, whereas UK Biobank uses hospitalization episode statistics (HES; encoded using ICD-10 clinical terms); (ii) All of Us is based on the U.S. population and U.S. health care system whereas UK Biobank is based on the UK population and UK health care system, which impacts diagnostic criteria and age at diagnosis; and (iii) All of Us individuals have different ancestries and socioeconomic backgrounds (including 26% African and 17% Latino; 78% of All of Us represents groups historically underrepresented in biomedical research based on race, ethnicity, age, gender identity, disability status, medical care access, income, and educational attainment) than UK Biobank individuals (94% European ancestry with higher than average income and educational attainment). We consider the cross-cohort prediction odds ratio of 1.32 to be an encouraging result given these key differences.

Comorbidity-based subtypes are genetically heterogeneous

We sought to verify that genetic differences between subtypes were not due to partitions of the cohort that are unrelated to disease (e.g. we expect a nonzero genetic correlation between tall vs. short type 2 diabetes cases, even if height is not genetically correlated to type 2 diabetes). Thus, we assessed whether the excess genetic correlation could be explained by non-disease-specific differences in the underlying topics (which are weakly heritable; Supplementary Table 3) by repeating the analysis using disease cases and controls with matched topic weights (i.e. case and controls have matched topic weights distributions within each disease or disease subtypes) (Methods). We determined that the excess genetic correlations could not be explained by non-disease-specific differences (Supplementary Fig. 20). We also estimated subtype-specific SNP-heritability and identified some instances of differences between subtypes, albeit with limited power (Supplementary Table 15).

Disease-associated SNPs have subtype-dependent effects

The third and fourth examples in Extended Data Fig. 8 are described here. Third, the hypertension-associated SNP rs3735533 within the *HOTTIP* long non-coding RNA has a lower odds ratio in the top quartile of CVD topic weight (1.07±0.02) than in the bottom quartile (1.13±0.02) ($P = 0.0015$ for interaction test ($FDR = 0.09 < 0.1$); P=0. 1 for top/bottom quartile test (FDR = 0.55)). *HOTTIP* is associated with blood pressure[4,5] and conotruncal heart malformations[6]. Fourth, the hypothyroidism-associated SNP rs9404989 in the *HCG26* long non-coding RNA has a higher odds ratio in the top quartile of FGND topic weight (1.90±0.24) than in the bottom quartile (1.19±0.13) ($P = 1\times10^{-4}$ for interaction test ($FDR = 0.02 < 0.1$); $P=3\times10^{-3}$for top/bottom quartile test (FDR = 0.15)). Hypothyroidism associations have been reported in the HLA region[4], but not to our knowledge in relation to the *HCG26*.

2 Additional discussion

Our findings reflect a growing understanding of the importance of context, such as age, sex, socioeconomic status and previous medical history, in genetic risk [7–9]. To maximise power and ensure accurate calibration, context information needs to be integrated into clinical risk prediction tools that combine genetic information (such as polygenic risk scores [10,11]) and non-genetic risk factors. Our work focuses on age, but motivates further investigation of other contexts. We note that aspects of context are themselves influenced by genetic risk factors, hence there is an open and important challenge in determining how best to combine medical history and/or causal biomarker measurements with genetic risk to predict future events[12].

We note several additional limitations. First, the genetic correlation and F_{ST} analyses were based on discrete subtypes, but discretizing continuous data loses information and may compromise power. However, definitions of disease often discretize continuous variables[13]. In addition, our PRS analysis (Fig. 6) and SNP x topic interaction analysis (Extended Data Fig. 8) leveraged continuous-valued topic weights. Second, interpretability can be a potential downside of data reduction approaches. The interpretation of a particular disease topic is that it consists of diseases that tend to co-occur with a specified set of diseases as a function of age. Identifying the functional biology underlying these co-occurrences remains a direction for future research, but there is immediate utility in performing disease subtype-specific GWAS and downstream analyses using the subtypes that we have identified.

3 Full Methods

Age-dependent topic model (ATM)

Our Age-dependent topic model (ATM) is a Bayesian hierarchical model to infer latent risk profiles for common diseases. The model assumes that each individual possesses several age-evolving disease profiles (topic loadings), which summarise the risk over age for multiple diseases that tend to co-occur within an individual's lifetime, namely the age specific multi-morbidity profiles. At each disease diagnosis, one of the disease profiles is first chosen based on individual weights of profile composition (topic weights), the disease is then sampled from this profile conditional on the age of the incidence.

We constructed a Bayesian hierarchical model to infer *K* latent risk profiles for *D* distinct common diseases. Each latent risk profile (comorbidity topics) is age-evolving and contains risk trajectories for all *D* diseases considered. Each individual might have a different number of diseases, while the disease risk is determined by the weighted combination of latent risk topics. The indices in this note are as follows:

- $s = 1,..., M$;
- $n = 1,..., N_s$;
- $i = 1,..., K$;
- $j = 1,..., D$;

where *M* is the number of subjects, N_s is the number of records within s^{th} subject, *K* is the number of topics, and *D* is the total number of diseases we are interested in. The plate notation of the generative model is summarised in Extended Data Fig. 1:

- $\theta \in R^{M \times K}$ is the topic weight for all individuals (referred to as patient topic weights), each row of which ($\in R^K$) is assumed to be sampled from a Dirichlet distribution with parameter α. α is set as a hyper parameter: $\theta_s \sim Dir(\alpha)$. We used topic weights to assign continuous values for disease subtypes in PRS and SNP x Topic analyses.

- $z \in \{1, 2,..., K\}^{\sum_s N_s}$ (referred to as diagnosis-specific topic probability) is the topic assignment for each diagnosis $w \in \{1, 2,..., D\}^{\sum_s N_s}$. Note the total number of diagnoses across all patients are $\sum_S N_S$. The topic assignment for each diagnosis is generated from a categorical distribution with parameters equal to s^{th} individual topic weight: $z_{sn} \sim Multi(\theta_s)$. We used diagnosis-specific topic probability to define discrete disease subtypes in excess genetic correlation and excess F_{ST} analyses.
- $\beta(t) \in F(t)^{K \times D}$ is the topic loading which is $K \times D$ functions of age t. $F(t)$ is the class of functions of t. At each plausible t, the following is satisfied: $\sum_j \beta_{ij}(t) = 1$. In practice we ensure above is true and add smoothness by constrain $F(t)$ to be a

softmax of spline or polynomial functions: $\beta_{ij}(t) = \frac{\exp(p_{ij}^T \phi(t))}{\{\sum_{j=1}^{D} \exp(p_{ij}^T \phi(t))}$, where $p_{ij}^T \phi(t)$ is polynomial and spline functions of t; $p_{ij} = \{p_{ijd}\}$; $d = 1, 2, ..., P$; P is the degree of freedom that controls the smoothness; $\phi(t)$ is polynomial and spline basis for age t.

- $w \in \{1, 2, ..., D\}^{\sum_S N_S}$ are observed diagnoses. The nth diagnosis of sth individual w_{sn} is sampled from the topic $\beta_{z_{sn}}(t)$ chosen by z_{sn}: $w_{sn} \sim Multi(\beta_{z_{sn}}(t_{sn}))$, here t_{sn} is the age of the observed age at diagnosis of the observed diagnosis w_{sn}.

The values of interest in this model are global topic parameter β, individual (patient) level topic weight θ, and diagnosis-specific topic probability z. Based on the generative process above, we notice that each patient is independent conditional on α. Therefore, the inference of θ and z(discussed below) is performed by looping each individual in turn.

The key element in our model is age-evolving risk profiles, which is achieved by model the comorbidity trajectories $\beta(t) \in F(t)^{K \times D}$ as functions of age. The functionals $F(t)$ are parameterized as linear, quadratic, cubic polynomials, and cubic splines with one, two and three knots. We use prediction odds ratio to decide the optimal model structure including the function forms and the number of topics; we use ELBO to choose the optimal inference results (with random parameter initialization) for the same model structure(Supplementary Table 1).

Inference of ATM

The variables of interest are global topic parameter $\beta(t)$, individual (patient) level topic weight θ, and diagnosis-specific topic probability z of each diagnosis. We could adopt an EM strategy, where in the E-step we first estimate posterior distribution of θ and z, then in the M-step we estimate β which maximises the evidence lower bound (ELBO).

The details of the inference is explained in the Analytic Note. In summary, in a Bayesian setting, We used the evidence function $p(w|\alpha, \beta)$ to evaluate how well the model fits the data. The best $\beta(t)$ is found by maximise the evidence function, while for θ and z we aim to find or approximate their posterior distribution $p(z, \theta \mid w, \alpha, \beta)$. Given that the posterior distribution is intractable, we use variational distribution $q(z, \theta)$ to approximate them. Now we could write the evidence function as:

$$p(w| \alpha, \beta) = L(z, \theta, \beta, \alpha) + KL(q||p),$$

here $KL(q||p) =- \int_{z,\theta} q(z, \theta) \ln \frac{p(z, \theta \mid w, \alpha, \beta)}{q(z, \theta)}$ is the KL divergence. Since KL divergence is always positive, $L(z, \theta, \beta, \alpha)$ is a lower bound of the evidence function:

$$L(z,\ \theta,\ \beta,\ \alpha)\ =E_q\{\ \ln p(w,\ z,\ \theta \mid \alpha,\ \beta)\ -\ \ln q(z,\ \theta\ )\}.$$

When finding the posterior of θ and z, we want ln $q(z,\ \theta\ )$ to be as close to the posterior $p(z,\ \theta \mid w,\ \alpha,\ \beta\ )$ as possible. Since $KL(q||p)\ =\ 0$ when $q(z,\ \theta\ )\ =\ p(z,\ \theta \mid w,\ \alpha,\ \beta\ )$, this is achieved by minimising $KL(q||p)$ or maximise $L(z,\ \theta,\ \beta,\ \alpha)$. The most commonly used form of $q(z,\ \theta\ )$ assumes the distribution is factorised, which might cause instability when signal-to-noise ratio is low[14]. Therefore, more accurate inference methods such as collapsed variational inference is considered[2]. Comparison of the evidence lower bound $L(z,\ \theta,\ \beta,\ \alpha)$ shows collapsed variational inference (CVB) is consistently more accurate than mean-field variational inference (VB) (Supplementary Fig. 7). Therefore we chose the collapsed variational inference[2]. The collapsed variational inference is achieved by integrate out θ from the likelihood function $p(w,\ z,\ \theta \mid \alpha,\ \beta)$ and find the approximated posterior distribution $q(z)$. For detailed derivation, the comparison between collapsed variational inference and mean-field variational inference, and update algorithms, see the Analytic Note.

When finding the $\beta(t)$ that maximises the evidence function, we again maximise $L(z,\ \theta,\ \beta,\ \alpha)$. Maximising $L(z,\ \theta,\ \beta,\ \alpha)$ with respect to $\beta(t)$ does not have an analytical solution due to its softmax structure. We use local variational methods and numeric optimisation to find the distribution of $\beta(t)$. In summary, L(z, θ, β, α) is not tractable with respect to $\beta(t)$ as it contains a log of softmax function (Section 3.2 of Analytic Note). We introduced a local variational variable to obtain a tractable lower bound of L(z, θ, β, α) (equation 11 in Supplementary Note) and use gradient descent to approximate the lower bound. Details are provided in the Analytic Note.

We extract topic weights at patient-level and diagnosis-level from the posterior distribution inferred from the data. Our model has the desired property that each patient and patient-diagnosis are assigned to comorbidity topics. The model estimates the posterior distribution $q(z)$, which is a categorical distribution (equation 8 of Analytic Note). We listed following definitions in this paper that are derived from the $q(z)$:

- Each patient-diagnosis (incident disease) has a *diagnosis-specific topic probability*, which is computed as $E_q\{z_n\}$.
- Each patient has a posterior *topic weights* θ_s, which is a dirichlet distribution $\theta_s \sim Dir(\ \alpha\ +\ \sum_{n=1}^{N_s}\ E_q\{z_n\}\ )$. The *topic weights* of each patient is defined as the mode of this Dirichlet distribution $\frac{\sum_{n=1}^{N_s} E_q\{z_n\}}{\sum_{i=1}^{K}\sum_{n=1}^{N_s} E_q\{z_{ni}\}}$ (we used $\alpha\ =\ 1$, which puts an noninformative prior on the topic weights). Topic weight is the low-rank representation of disease history, for analyses including PRS association with comorbidity topics and SNP x Topic interaction analysis.

- The *average topic assignments* of disease j is the mean over all incidences $\overline{E_q\{z_{sn \in \{w_{sn}=j\}}\}}$. This metric is used to measure which comorbidity topic a disease is associated with (Fig. 4B), and it is equivalent to a weighted average of topic loadings (Supplementary Note equation 5 shows the link between diagnosis-specific topic probability and topic loading). A disease assigned to multiple topics is considered to have comorbidity subtypes.
- A hard assignment of a patient-diagnosis to a *comorbidity-derived subtype* is based on the max value of the vector $E_q\{z_n\}$. The incident disease is assigned to topic $argmax_i\,(E_q\{z_{ni}\})$.

Metrics for evaluating ATM

ATM is evaluated for different purposes, which requires different metrics (Supplementary Table 1). Here we list the details of the four metrics considered: *Prediction odds ratio, Evidence Lower Bound (ELBO), the Area under the Precision-Recall curve (AUPRC)*[15]*, and Co-occurrence odds ratio.*

Prediction odds ratio: We used prediction odds ratio to compare models of different topic numbers and configuration of age profiles. Briefly, prediction odds ratio is defined on 20% held-out test data as the odds that the true diseases are within the top 1% diseases predicted by ATM (trained on 80% of the training set and uses earlier diagnoses as input), divided by the odds that the true diseases are within the top 1% of diseases ranked by prevalence.

Specifically, we separate UK Biobank patients into a training set (80%) and a testing set (20%). On the training set, we estimate the comorbidity topic loadings. On the testing set, we fix the topic loadings and infer the patient topic weights to predict the next disease in chronological order. The topic loadings are estimated using the n diseases and compute the risk rank of diseases at the age of the n+1 disease. The odds ratio is computed by the odds of the n+1 disease being in the top 1% of diseases versus being in the top 1% most prevalent diseases. We use the top 1% most prevalent diseases instead of randomly chosen diseases as it represents a naive prediction model that predicts disease based on prevalence. The patient topic weights computation is in section Inference of ATM and the risk is computed as the linear combination of topics using topic weights as coefficients. We also compute the prediction odds ratio using the LDA model. We repeat the procedure for 10 times for each model configuration.

We compared the prediction odds ratio for fitting UK Biobank with ATM of varying topic numbers (5 to 20) and age-dependent functions (linear, quadratic polynomial, cubic polynomial, and splines with one, two and three knots). We also compare the ATM model with the LDA model of topic number between 5 to 20.

Evidence Lower Bound (ELBO): ELBO evaluated the accuracy of the variational inference method on a specific data set [1]. The mathematical expression of ELBO for ATM is presented

in equation 9 in the Analytic Note. To find the best model that fits the entire dataset, we evaluate the ELBO for models with 19 choices of the number of topics: 5-20, 25, 30, and 50; 6 choices of age profiles configuration: linear, quadratic polynomial, cubic polynomial, and splines with one, two and three knots. Each model is run for 10 times with random initialisations. We choose the model that has the highest ELBO after converging.

AURPC: To evaluate whether a model could capture the comorbidity subtypes in simulation analysis, we compute the precision, recall, and area under precision-recall curve (AUPRC) to correctly classify disease diagnosis to be from the topic that it is generated from. The topic of each diagnosis is determined by diagnosis-specific topic probability. Note we could only evaluate AUPRC in simulations where the truth is known.

Co-occurrence odds ratio: To verify that the comorbidity profiles that the model captured are capturing diseases that are more likely to present within the same individual, we estimate the odds ratio of the disease duo, trio, quartet, and quintet that are captured by the topic versus that of random combinations. We divide the population into an 80% training set and a 20% testing set. We trained the ATM model with five random initialisations and kept the model with the highest ELBO. Each disease is assigned to a topic by the highest average topic assignments. (section Inference of ATM) We focus on the top 100 diseases ranked by prevalence to avoid the combination being too rare to appear in the population. In the testing set, we computed the odds of individuals who have all diseases in the comorbidities versus the odds implied if all diseases are independent (computed as the product of disease prevalence). The odds ratio is computed for all combinations of duo, trio, quartet, and quintet that are assigned to the same topics. We perform the same analysis using PCA for comparison.

Simulations of ATM method

To test whether the algorithm could assign diagnoses to correct comorbidity profiles, we simulated diagnoses from two comorbidity profiles (topics) in a population of 10,000, using following parameters:

- $M = 10,000$;
- $\overline{N_S} = 6.1$;
- $N_S \sim \exp\{\overline{N_S}\}$;
- $D = 20$;
- $K = 2$;

Here M is the number of individuals in the population, $\overline{N_S}$ is the average number of diseases for each individual, D is the total number of diseases, K is the number of comorbidity topics. The distribution of disease number per-individual N_S is sampled from an exponential distribution, which matches those from UK Biobank data (Supplementary Fig. 25). According to equation 3.1 in Ghorbani et al.[14], whether the topic model could capture the true latent structure is determined by the information signal-to-noise ratio and could be evaluated

with limits $M \rightarrow \infty$; $D \rightarrow \infty$; $\frac{D}{M} \rightarrow \delta$, where δ is a constant. Therefore we choose D and M that make $\frac{D}{M}$ similar to those of the UK Biobank dataset (Samples size = 282,957; distinct disease number = 349).

The simulated topics loadings are constructed as follows:

- All but K diseases are simulated to be associated with comorbidity profiles. Each of them has a risk period of 30 years and overlaps for 10 years with the next disease. For example, if disease 1 has a risk period from 30 to 59 years of age, disease 2 will have a risk period between 50 to 79 years of age. When the risk period reaches the maximal age, the truncated part will be carried to the next disease to create diseases with shorter risk period. All risk periods are assigned a value 1. The overlapping structure of topic loadings is chosen so that average standard deviation in age-at-diagnosis (8.5 years) and the age window under consideration (30-80 years of age) matches UK Biobank data.
- K diseases that are not associated with comorbidity are simulated to span all topics. The values of these diseases are sampled from $Unif(0, \frac{0.1}{K})$ for each topic. Here K is the number of topics.
- The age profiles are then normalised at each age point to ensure $\sum_{j=1}^{D} \beta_j(t) = 1$ for all t. With this constraint we could sample a disease at each age t using a multinomial probability with the topic loading as the parameter. The age range of the simulated topics is 30 to 81 years of age, which is the minimal and maximal age at diagnosis of incident disease in the UK Biobank population. An example of a simulated topic is shown in Supplementary Fig. 26.

For each individual, we sampled the Dirichlet parameter α from a gamma distribution (shape = 50, rate = 50). Topic loadings are sampled from the Dirichlet distribution for each patient as the generative process. For each patient, we first sample the number of diseases N_S. For each incident disease, we sample the disease age from uniform distribution between age 30 to 81 and a topic from the topic loading. We then choose the incident disease based on the age at diagnosis from the chosen topic. The procedure follows the generative process described above.

Since in real data we only use the first age at diagnosis for recurrent diseases within the same patient, we filter the simulated diseases accordingly. The filtered data are fed into the inference functions to infer the latent topics and disease assignments. The inferred topics compared with the true topics used to simulate diseases are shown in Supplementary Fig. 26. For the initialisation of each inference, we first sample β and θ from the Dirichlet distribution of non-informative hyperparameters, then initialised other variables parameters following the generative process. The variational inference converged where the relative increase of ELBO is below 10^{-6}.

We simulated diseases with distinct comorbidity subtypes by combining diseases from distinct topics and labelling them as a single disease, using parameters described above. We consider two scenarios: (1) the subtype of diseases have the same age at diagnosis distribution. (2) the subtypes of disease have distinct age at diagnosis distribution. We first chose one disease (**disease A**) then sampled a proportion of a second disease (**disease B**) to label as **disease A**. The proportion is varied to create a different sample size ratio of the two subtypes. In scenario one, **disease B** is a disease that has the exact same age distribution as **disease A** but from the other topic. In scenario two, **disease B** is from the other topic and has a different age distribution (age at diagnosis moves up for 20 years, 10 years, or 5 years, respectively) than **disease A**. After changing the labels of **disease B** to be the same as **disease A**, we used the inference procedure described as above to get the posterior distribution.

To evaluate whether a model could capture the comorbidity subtypes, we compute the precision, recall, and area under precision-recall curve (AUPRC) of correctly classifying incident **disease B** to be from the topic that it is generated from. The topic of each diagnosis is determined by diagnosis-specific topic probability. We use other diseases from the topic of **disease B** to benchmark the topic label. Topic modelling on the simulated data is performed with both ATM and LDA (both implemented using collapsed variational inference for fair comparison) to compare the performances.

We evaluate the subtype classification with varying values for four simulation parameters:

- ratio of sample sizes between the two subtypes. We change the ratio of the two subtypes by a grid between 0 to 0.9 with a step size 0.1. The default value of sample size ratio is set as 0.1 in other simulations to test for other parameters that have impacts on the precision and recall.
- Simulated population size. We simulated population sizes equal to 200, 500, 1000, 2000, 5000, and 10,000. The default population size is 10,000 in other simulations.
- Number of distinct diseases. We simulated datasets with 20, 30, 40, and 50 distinct diseases, with 2, 3, 4 and 5 underlying disease topics respectively. The default number of distinct diseases is 20 in other simulations.
- Difference of age distribution. We considered three scenarios of subtype age distribution, with 0, 10, and 20 years of difference in the average age at diagnosis.

UK Biobank comorbidity data

We analysed comorbidity data from 282,957 UK Biobank samples with diagnoses for at least two of the 348 focal diseases that we studied (see below). We use the hospital episode statistics (HES) data within the UK Biobank dataset, which records diseases using the ICD-10/ICD-10CM coding system; the average record span of HES data is 28.6 years. Codes started with letters from A to N are kept as they correspond to disease code (opposed to procedure codes). The disease records were mapped from ICD-10/ICD-10CM codes to PheCodes using a three-step procedure: First, we mapped the first four letters of each ICD-10 records to the phecodes, using the map file downloaded from phewascatalog.org; second, we

mapped the remaining records using ICD-10CM map file downloaded from phewascatalog.org; last, we mapped remaining records using the same ICD-10CM map system but only use the first four character of each ICD-10CM codes. We also noticed (ICD-10/ICD-10CM)-Phecode pairs are not always one-to-one; when a single ICD-10/ICD-10CM code is mapped to more than one PheCodes, we chose the Phecode with the largest number of links to ICD-10/ICD-10CM codes to reduce redundancy of the mapping result. Using the procedure above, we mapped 99.7% ICD-10/ICD-10CM code to PheCodes, with 4,637,127 records in total.

The mapped Phecodes are filtered to keep only the first age at diagnosis for the same diseases within a patient. The age at diagnosis for each record is computed as the difference between month of birth to the episode starting date. We then computed the occurrence of each disease in the UK Biobank and kept 348 that have more than 1,000 occurrences (Supplementary Table 4). Starting with all 488,377 UK Biobank patients (including both European and non-European ancestries), we filtered the patients to keep only those who have at least two distinct diseases from the 348 focal diseases, as we are most interested in the comorbidity information. We treated the death as an additional disease (8,666 records) to evaluate if certain comorbidities are more likely to lead to fatal events. After these procedures, there are in total 1,726,144 distinct records across 282,957 patients.

To name the topics inferred from the UK Biobank, we take the sum of *average topic assignments* (section Inference of ATM) over diseases for each Phecode system and extract the three most common Phecode disease systems. Six topics are named using the three most common Phecod disease systems: NRI “neoplasms, respiratory, infectious diseases”, CER “cardiovascular, endocrine/metabolic, respiratory”, SRD “sense organs, respiratory, dermatologic”, FGND “female genitourinary, neoplasms, digestive”, MGND “male genitourinary, digestive, neoplasms”, MDS “musculoskeletal, digestive, symptoms”. For four topics that are predominantly associated with one system, we name them based on their top associated Phecode system: LGI “lower gastrointestinal”, UGI “upper gastrointestinal”, CVD “cardiovascular”, and ARP “arthropathy”.

We present topic loadings of a few focal diseases in two ways. Firstly, we filter each topic using the profile mean value between age 30 to 81 to keep the top seven diseases. We chose seven for visualisation, as we found more diseases would be harder to read on a plot. Secondly, we also show seven diseases that have the highest *average topic assignment* to each topic. This will give a picture of diseases that are not the most prevalent in the population but are predominantly associated with the target topic.

To compare the comorbidity heterogeneity between age groups, we group the incidences for each disease to two age groups: young group (<60 years of age) and old group (≥60 years of age). We compute the average topic assignment of each group as described in section Inference of ATM. Additionally, we inferred topics for male (984,554 records in 156,366 individuals) and female (741,590 records in 126,591 individuals) populations respectively

using a model with 10 topics and spline function with one knot. We extract the average topic assignment for each disease, and use Pearson's correlation to match the topics for both sexes to the topics inferred on the entire population.

We assigned diagnoses to discrete subtype using max diagnosis-specific topic probability. We focus our genetic heterogeneity analysis on 52 diseases that have at least 500 incidences assigned to a secondary topic.

All of Us comorbidity data.
We analysed EHR data collected in the EHR domain of All of Us samples, which includes both primary care and secondary care data. The average distance between first and last diagnoses is 7.9 years (vs. 7.0 years in UK Biobank); the average record span period is unknown, but we hypothesized that it is likely to be considerably larger than 7.9 years (vs. 28.6 years in UK Biobank). Disease codes in the All of Us EHR domain are coded in SNOMED CT. We first mapped All of Us disease codes from SNOMED CT to ICD-10CM code using map version 20220901 downloaded from https://www.nlm.nih.gov/research/umls/mapping_projects/snomedct_to_icd10cm.html. When a single SNOMED CT code was mapped to multiple ICD-10CM codes, we choose the code with the highest UK Biobank prevalence from these ICD-10CM codes. We then mapped ICD-10CM codes to Phecodes, using the same procedure described in the section above. We kept 233 Phecodes that overlap with the 348 diseases analysed in the UK Biobank. We kept the first diagnosis for recurrent diseases in each patient. After mapping, we are left with 3,098,771 diagnoses spanning 211,908 All of Us samples. We run ATM with topic number from 5 to 20 and spline with two knots (degree of freedom = 5) on the All of Us comorbidity data and computed prediction odds ratio (using five-fold cross validation) and ELBO (on all 211,908 samples).

Comparing disease topics between UK Biobank and All of Us
We compared the optimal models from UK Biobank (10 topics, degree of freedom = 5) and All of Us (13 topics, degree of freedom = 5). We constrained our analyses on 233 of the 348 diseases that are shared between the two data sets. We performed three analyses to compare the comorbidity patterns from the two data sets.

First, we computed the correlation of topic loadings from two data sets. Since the topic loadings are functions of age, we computed their correlations using four different ways to summarise age information: topic loadings averaged across age; topic loadings at age 50, 60, and 70. For each UK Biobank topic, we found its most similar All of Us topic that has max correlation of topic loadings (averaged across age).

Second, we computed the cross-population prediction odds ratio, using the All of Us topics to predict on UK Biobank comorbidity data. We divided the UK Biobank samples into 10 jackknife blocks and computed prediction odds ratios on each leave-one-out sample.

Third, we compared the correlation of comorbidity profiles (measured by average topic assignments; see Methods for definition) for 233 diseases that are shared between the two populations. We define *correlations between topic assignments* as the correlation between UK Biobank average topic assignments and All of Us average topic assignments after mapped to UK Biobank topic space (see below).

Comparing disease topics inferred from different data sets is challenging due to the exchangeability of topics (i.e. distinct topic configurations have the same likelihood for a given data set). To compute *correlations between topic assignments* from ATM inference on different populations, we first mapped the topics to the same topic space. Suppose there are two topic spaces $\{T^1\}$ and $\{T^2\}$. We create a map $s(.)$ from $\{T^1\}$ and $\{T^2\}$ by computing the normalised R^2 between topic loadings:

$$s(\{T^1\},\ \{T^2\})_{i1,i2} = \frac{cor(T^1_{i1}, T^2_{i2})_+^{\ 2}}{\sum_{k2}^{K_2} cor(T^1_{i1}, T^2_{k2})_+^{\ 2}};$$

here T^1_{i1} is the $i1^{th}$ topic loading from $\{T^1\}$, T^2_{i2} is the $i2^{th}$ topic loading from $\{T^2\}$; K_1, K_2 is the number of topics in $\{T^1\}$, $\{T^2\}$; $cor(.)_+$ is the positive part of the correlation, where we set the negative correlations to zero as negative correlations between topic loadings are uninformative consequences of the multinomial distribution in the model. Intuitively, $s(\{T^1\},\ \{T^2\})_{i1,i2}$ maps each T^1_{i1} to $\{T^2\}$ based on the proportion of T^1_{i1} variance explained by the K_2 topics in $\{T^2\}$. $s(\{T^1\},\ \{T^2\})\in R^{K_1\times K_2}$.

Suppose we have the comorbidity profile of disease 1 and disease 2, which lies in $\{T^1\}$ and $\{T^2\}$ respectively. We could map disease 1 diagnosis-specific topic probability $z_{sn,1}\in R^{K_1}$ (or average topic assignments of disease 1; see these definitions in Methods) to topic space 2: $z'_{sn,1} = s(\{T^1\},\ \{T^2\})^T z_{sn,1}$, $z'_{sn,1} \in R^{K_2}$. The correlations between topic assignments in topic space 2 between disease 1 and disease 2 is the correlation $cor(\overline{z'_{sn,1}}, \overline{z_{sn,2}})$; here $\overline{z'_{sn,1}}$ is the average topic assignments for disease 1 after mapped to topic space 2, which is the average of $z'_{sn,1}$ across all diagnoses; $\overline{z_{sn,2}}$ is the average topic assignments for disease 2. For correlations between topic assignments within the same topic, $s(\{T^1\},\ \{T^1\})$ is an identical matrix.

UK Biobank genotype data.
For genetic correlation analysis, F_{ST}, and SNP x Topic interaction analyses, we used genetic data from 488,377 UK Biobank participants (prior to restricting to 282,957 samples with at least two of the 348 diseases studied). For PRS and heritability estimation of the 10 topics, we constrained our analysis to 409,694 British Isle ancestry individuals to adjust for

population structure; we used the mixed-effect association model implemented in BOLT-LMM software[16,17] to adjust for population structure. For F_{ST} analysis with PLINK we used 805,426 genotyped SNPs; for BOLT-LMM PRS analysis we used 727,882 genotyped SNPs with MAF>0.1%. For genetic correlation analysis using LDSC, we used 157,756 Genotyped SNPs mapped to HapMap3 SNPs. For computing heritability, we used the mixed-effect association in BOLT-LMM [16,17] to generate summary statistics, and used LDSC[18] to estimate heritability, where we used 1,201,838 imputed SNPs mapped to HapMap3 SNPs SNPs.

Polygenic risk scores (PRS) analysis.
Despite population stratification cannot be excluded[19], to adjusted for and minimize the impact of population stratification, we applied mixed-effect association model to samples of British Isle ancestry group (N = 409,694) to compute PRS, for 10 heritable diseases that have the highest heritability z-scores. We used a mixed model to estimate effect size implemented by BOLT-LMM and constructed genome-wide PRS [17]. We sample controls to keep a balanced ratio of case and controls. For four diseases with more than 20,485 case (essential hypertension, arthropathy, asthma, and hypercholesterolemia), we downsampled controls to make the total sample size half of that of British isle ancestry population (N = 204,847) for computation efficiency; for other diseases, we sampled 9 controls for each case to ensure case proportion at or above 10% as recommended by BOLT-LMM (type 2 diabetes, varicose veins of lower extremity, hypothyroidism, other peripheral nerve disorders, major depressive disorder, and GRED). We used PLINK to select genotyped SNPs with MAF > 0.1% as recommended in BOLT-LMM. For each disease, we used 5-fold cross validation to estimate effect sizes using BOLT-LMM and computed predictive PRS on the held-out testing set. We used linear regression between continuous-valued topic weights and the predictive PRS to compute the excess PRS over different topics, where PRS is the response variable and topic weights is the predictor.

We compute the subtype-specific relative risk for each percentile of PRS using the following formula:

$$RR_{pt,s} = \frac{n_{pt,s} \times 100}{n_s},$$

where $RR_{pt,s}$ is the relative risk of s subtype for the pt^{th} PRS percentile (computed for the entire population); $n_{pt,s}$ is the number of cases in s subtype that has PRS within the pt^{th} percentile; n_S is the number of cases in the s subtype.

Genetic correlation analysis.
We used discrete subtypes in genetic correlation analysis (different from the PRS analysis above). For each disease and disease subtype, we use a case-control matching strategy to construct data to estimate coefficients for genetic correlation analysis. For each case in the disease group, we pick four nearest neighbors (without replacement) from the control group, matching sex, BMI, year of birth and 40 genetic principal components. The covariates are

available within the UK Biobank data set, over which we computed the principal components. We then compute the Euclidean distance of the principal components to find the nearest neighbours in the population. All cases are matched with four controls except for 401.1 essential hypertension which has a sample size larger than 20% of the population. We match only one control for each hypertension case.

We perform logistic regression with sex and top 10 principal components as covariates to estimate the main variant effect of the 805,426 variants that are genotyped. We used PLINK 1.9 for association analysis[20]. With the summary statistics from the association analysis, we use LDSC to map the summary statistics to HapMap3 SNPs and match the effect and non-effect alleles[18,21]. Since UK Biobank is mostly of British Isle ancestry, we use the pre-computed LD score from the LDSC website. We estimated the heritability for each disease or disease subtype which has more than 1000 incidences (378 = 30 diseases subtypes + 348 diseases). We use 1000 incidence threshold as LDSC are more accurate with larger sample size. We focus on 71 disease and 18 disease subtypes of the 378 diseases subtypes and diseases that have heritability z-score above 4 for genetic correlation analysis.

The genetic correlation is computed for each pair of disease-disease, disease-subtype, and subtype-subtype using the logistic regression summary statistics and LD score regression. We report the estimate of genetic correlation and z-scores. Additionally, for pairs that involve subtypes (disease-subtype or subtype-subtype), we compute the excess genetic correlation, defined as the difference between the genetic correlation involving subtypes (disease-subtype and subtype-subtype) and the genetic correlation involving all disease diagnoses (disease-disease). For example, the genetic correlation between T2D-CER and hypertension-CVD is compared to the genetic correlation between all T2D and all hypertension. The z-score and p-value of the genetic correlation differences are reported. We note that genetic correlations between subtypes of the same disease are compared to 1. We only reported p-values of excess genetic correlation when both genetic correlation estimation has standard error <0.1 and at least one of the genetic correlation has |z-score|>4.

To avoid potential collider effects where subtypes are defined by topic components that are independent of the diseases, we performed the same genetic correlation analyses but match cases in each subtype with controls with similar topic loadings. We computed PCs from 23 variables (10 topic loadings, 10 PCs, year of birth, sex, and BMI) and used the nearest neighbour procedure (by Euclidean Distance) to find controls for each case. Here controls are chosen from individuals without the targeting disease, i.e. an individual with one subtype of the target disease could not be a control for the other subtypes. We performed the same analysis using this case-control matching procedure and compared the genetic correlation with the case-control procedure described above. We perform the analysis for four diseases that have evidence for genetic subtypes: asthma, type 2 diabetes, hypercholesterolemia, and hypertension. For one subtype (hypertension-CVD), the heritability (0.0313, s.e. = 0.0289) is below threshold after matching the topic, which was excluded in genetic correlation analysis.

F_{ST} analysis.

We used discrete subtypes in genetic correlation analysis (same as genetic correlation analysis above; different from the PRS analysis). To evaluate the genetic heterogeneity between disease subtypes, we estimated the F_{ST} for 52 diseases that have at least 500 incidences assigned to a secondary topic. To test the statistical significance of Fst, we adopted a permutation strategy by sampling controls with matched topic weights and sample size for each disease subtype, and computed F_{ST} across the subtype-matched control groups. For each disease subtype, we match the topic weights of permutation samples by sampling (without replacement) the same number of controls as the cases for each quartile of topic weight that defines the subtype, which ensures the permutation null samples have the same topic weight stratification as the disease subtypes. We then compute the F_{ST} across the control groups (each group is matched with one disease subtype) for each disease. We excluded three diseases, “hypertension”, “hypercholesterolemia”, and “arthropathy”, from F_{ST} analysis as we do not have enough controls that match topic weight distribution. The F_{ST}s are computed using PLINK 1.9's weighted mean across all genotyped SNPs, which report F statistics across all subtypes.

We obtained 1,000 permutation samples and reported the permutation p-value. Under the assumption that causal and non-causal variants have similar allele frequency differences across the subtypes, F_{ST} is a measure of causal genetic effect heterogeneity across subtypes.

SNP x topic interaction test.
We used continuous-valued topic weights in the SNP x topic interaction analysis (same as the PRS analysis; different from the genetic correlation and F_{ST} analyses). For the diseases that have heritability z-score above 4 in the UK Biobank, we further investigated whether there are interactions between genetic risk factors with the topic loadings. We used a fit a logistic regression model using following model:

$$logit(p) = \beta_0 + \beta_1 * T + \beta_2 * T^2 + \beta_3 * G + \beta_4 * G * T,$$

where T is individual topic weights for a specified topic, G is the genotype, and p is the probability of getting the disease. We computed the test statistics under the null that $\beta_4 = 0$.

Since the simulation shows the interaction test is underpowered when the variant effects are small, we focus on the set of SNP that reaches genome-wide significance level to increase power to detect interaction effects. We performed LD-clumping using $r^2 > 0.6$ to remove variants that are in strong LD with the lead variants. We computed the test statistics using the model above (for testing $\beta_4 = 0$) and computed study-wise FDR across 2530 disease-topic pairs.We used QQ plots to check that interaction test statistics computed using all non-subtype topics for each disease (which are expected to be null) were well-calibrated. (Supplementary Fig. 24B).

As an alternative way to verify the interactions, we divided cases into quartiles based on topic weights (which defines disease subtypes continuously) for each disease-topic pair, and randomly sampled two controls that match the topic weights for each case. We estimated the

main effect sizes for all GWAS SNPs within each quartile of topic weight and compared the effects between the top and bottom quartiles of topic weights. For visualisation, we use GWAS SNPs that have no interaction effect (above, P>0.05) as background SNPs.

Simulations of SNP x topic interaction

We simulate comorbidity with genetics to test interaction between genetic and comorbidity topics. We simulated 100 independent variants with MAF randomly sampled from the MAF of 888 independent disease associated SNPs. We assumed an additive model and simulated genotypes for the population using Hardy-Weinberg equilibrium. We simulated three types of genetic effects on topic and diseases on topic of the simulation framework described in Simulations of ATM method section:

- Genetics-topic effect: each variant is simulated to have an linear effect of 0.04 on the topic loading. We choose this value as after normalising the topic, a regression of causal variant to topic would have an effect size approximately 0.01 which is similar to our observation in the UK Biobank. The number of variants that are causal to the topic varies between 2 to 20. We simulated the effect on one topic by adding additive SNP effects and normalise the topic loadings of each patient. The topic-disease causality is a natural consequence following the generative process of sampling data.
- Genetic-disease-topic effect: we simulated a heritable disease that is causal to the topic. The disease is simulated with 20 causal variants each of effect size 0.15. We vary the disease-to-topic causal effect from 0.05 to 0.5, with a default value of 0.1 in other analyses (similar to the correlation we found in UK Biobank analysis). We simulated the effect on one topic by adding additive causal disease effects and normalise the topic loadings of each patient.
- The genetic effect could interact with the topic when contributing to disease risk. We simulated four additional diseases to represent different structures (Supplementary Fig. 21).
    - Genetic effects interact with topic loading on altering disease risk. The interaction term is added to the mean of disease liability, which is sampled from a Gaussian distribution. The disease is then sampled by a threshold on the liability, where the incidence rate is by default 0.5. The interaction effect is varied from 0.4 to 4, with default value equal to 2.
    - Pleiotropy effects are simulated with a variant that have both genetic-disease and genetic-topic-disease effects. Both genetic and topic effects are added to the mean of disease liability. A disease is sampled by a threshold with default incidence rate equal to 0.5. The topic-disease effect is varied from 0.4 to 4, with default value equal to 2.
    - Pleiotropy effect with nonlinear topic-disease effect. A quadratic term of topic-disease effect added to the second model.
    - Pleiotropy effect with nonlinear genetic-disease effect. A quadratic term of genetic-disease effect added to the second model.

We simulated with varying disease-topic or topic-disease causal effects with 50 repetition at each causal effect size to obtain uncertainty quantification. The simulated data are fed to the

ATM to infer the topic weights for interaction testing. The test statistics are for a the interaction (null $b_3 = 0$ in Supplementary Fig. 21-22) between topic weights and genotypes.

References for full methods

1. *Bishop, C. M. Pattern Recognition and Machine Learning. (Springer New York, 2006).*
2. *Teh, Y., Newman, D. & Welling, M. A collapsed variational Bayesian inference algorithm for latent Dirichlet allocation. Adv. Neural Inf. Process. Syst.* ***19****, (2006).*
3. *Blei, Ng & Jordan. Latent dirichlet allocation. J. Mach. Learn. Res. (2003).*
4. *Sakaue, S. et al. A cross-population atlas of genetic associations for 220 human phenotypes. Nat. Genet.* ***53****, 1415–1424 (2021).*
5. *Kanai, M. et al. Genetic analysis of quantitative traits in the Japanese population links cell types to complex human diseases. Nat. Genet.* ***50****, 390–400 (2018).*
6. *Oluwafemi, O. O. et al. Genome-Wide Association Studies of Conotruncal Heart Defects with Normally Related Great Vessels in the United States. Genes* ***12****, (2021).*
7. *Jiang, X., Holmes, C. & McVean, G. The impact of age on genetic risk for common diseases. PLoS Genet.* ***17****, e1009723 (2021).*
8. *Mostafavi, H. et al. Variable prediction accuracy of polygenic scores within an ancestry group. Elife* ***9****, e48376 (2020).*
9. *Dumitrescu, L. et al. Evidence for age as a modifier of genetic associations for lipid levels. Ann. Hum. Genet.* ***75****, 589–597 (2011).*
10. *Chatterjee, N., Shi, J. & García-Closas, M. Developing and evaluating polygenic risk prediction models for stratified disease prevention. Nat. Rev. Genet.* ***17****, 392–406 (2016).*
11. *Abul-Husn, N. S. & Kenny, E. E. Personalized Medicine and the Power of Electronic Health Records. Cell* ***177****, 58–69 (2019).*
12. *Lin, J. et al. Integration of biomarker polygenic risk score improves prediction of coronary heart disease in UK Biobank and FinnGen. bioRxiv (2022) doi:10.1101/2022.08.22.22279057.*

13. *Falconer, D. S. The inheritance of liability to diseases with variable age of onset, with particular reference to diabetes mellitus. Ann. Hum. Genet.* ***31****, 1–20 (1967).*

14. *Ghorbani, B., Javadi, H. & Montanari, A. An Instability in Variational Inference for Topic Models. in Proceedings of the 36th International Conference on Machine Learning (eds. Chaudhuri, K. & Salakhutdinov, R.) vol. 97 2221–2231 (PMLR, 09--15 Jun 2019).*

15. *Davis, J. & Goadrich, M. The relationship between Precision-Recall and ROC curves. in Proceedings of the 23rd international conference on Machine learning 233–240 (Association for Computing Machinery, 2006).*

16. *Loh, P.-R. et al. Efficient Bayesian mixed-model analysis increases association power in large cohorts. Nat. Genet.* ***47****, 284–290 (2015).*

17. *Loh, P.-R., Kichaev, G., Gazal, S., Schoech, A. P. & Price, A. L. Mixed-model association for biobank-scale datasets. Nat. Genet.* ***50****, 906–908 (2018).*

18. *Bulik-Sullivan, B. et al. An atlas of genetic correlations across human diseases and traits. Nat. Genet.* ***47****, 1236–1241 (2015).*

19. *Haworth, S. et al. Apparent latent structure within the UK Biobank sample has implications for epidemiological analysis. Nat. Commun.* ***10****, 333 (2019).*

20. *Chang, C. C. et al. Second-generation PLINK: rising to the challenge of larger and richer datasets. Gigascience* ***4****, 7 (2015).*

21. *International HapMap 3 Consortium et al. Integrating common and rare genetic variation in diverse human populations. Nature* ***467****, 52–58 (2010).*

4 Analytical Notes

4.1 Usage instruction for the analytical notes

The purpose of this supplementary note is to provide a self-contained explanation of the mathematical basis underlying our topic based model. Therefore, the materials are not meant to provide new discoveries but to help readers to derive all of our inference methods without referring to external materials (though we do listed references to text wherever appropriate). As a consequence, we made no efforts to condense steps, and opt to expand with more details when we feel it is necessary.

4.2 Generative process of a curve topic model

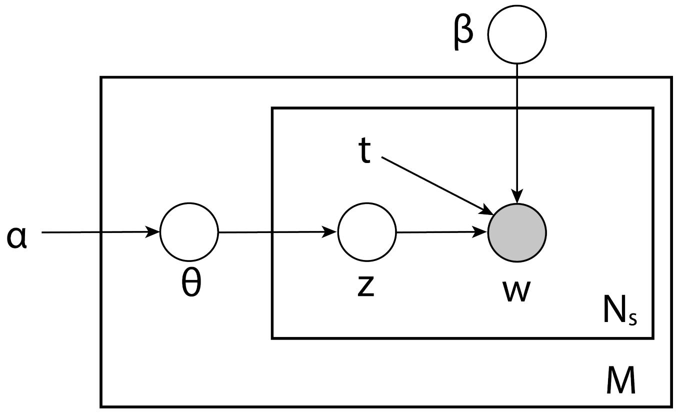

Supplementary Figure 1: Plate notation of ATM generative model. M is the number of subjects, N_s is the number of records within s^{th} subject. All plates (circles) are variables in the generative process, where the plates with shade w is the observed variable and plates without shade are unobserved variables to be inferred. θ is the topic weight for all individuals; z is diagnosis-specific topic probability; t is the age at onset for each diagnosis; β is the topic loadings which are functions of age t; α is the (non-informative) hyperparameter of the prior distribution of θ. The generative process is described in the Methods and Supplementary Note.

We constructed a Bayesian hierarchical model to infer latent risk profiles for common diseases. In summary, the model assumes there exist a few disease topics that underlie many common diseases. Each topic is age-evolving and contain risk trajectories for all diseases considered. An individual's risk for each diseases is determined by the weights of all topics. The indices in this note are as follows:

$$s = 1, ..., M;$$

$$n = 1, ..., N_s;$$
$$i = 1, ..., K;$$
$$j = 1, ..., D;$$

where M is the number of subjects, N_s is the number of records within s^{th} subject, K is number of topics, and D is the total number of diseases we are interested in. The generative process (Supplementary Figure 1) is as follows:

- $\theta \in \mathcal{R}^{M\times K}$ is the topic weight for all individuals, each row of which ($\in \mathcal{R}^K$) is assumed to be sampled from a Dirichlet distribution with parameter α. α is set as a hyper parameter.

$$\theta_s \sim Dir(\alpha).$$

- $\mathbf{z} \in \{1, 2, ..., K\}^{\sum_s N_s}$ is the topic assignment for each diagnosis $\mathbf{w} \in \{1, 2, ..., D\}^{\sum_s N_s}$. Note the total number of diagnoses across all patients are $\sum_s N_s$. The topic assignment for each diagnosis is generated from a multinoulli distribution with parameter equal to s^{th} individual topic weight.

$$z_{sn} \sim Multi(\theta_s).$$

- $\beta(t) \in \mathcal{F}(t)^{K\times D}$ is the topic which is $K \times D$ functions of age t. $\mathcal{F}(t)$ is the class of functions of t. At each plausible t, the following is satisfied:

$$\sum_j \beta_{ij}(t) = 1.$$

In practice we use softmax function to ensure above is true and add smoothness by constrain $\mathcal{F}(t)$ to be spline or polynomial functions:

$$\beta_{ij}(t) = \frac{\exp(\boldsymbol{p}_{ij}^T \phi(t))}{\sum_{j=1}^{D} \exp(\boldsymbol{p}_{ij}^T \phi(t))},$$

where $\boldsymbol{p}_{ij} = \{p_{ijd}\},\ d = 1, 2, ..., P$; P is the degree of freedom than controls the smoothness; $\phi(t)$ is polynomial and spline basis for age t.

- $w \in \{1, 2, ..., D\}^{\sum_s N_s}$ are observed diagnoses. The nth diagnosis of sth individual w_{sn} is sampled from the topic $\beta_{z_{sn}}(t)$ chosen by z_{sn}:

$$w_{sn} \sim Multi(\boldsymbol{\beta}_{z_{sn}}(t_{sn})),$$

here t_{sn} is the age of the observed age-at-onset of the observed diagnosis w_{sn}.

The value of interest in this model are global topic parameter β, individual (patient) level topic value θ, and topic value z of each diagnosis. Based on the generative process above, we notice each patient is independent conditional on α and β. Therefore, we could adopt an EM strategy, where we first estimate θ and z, then estimate β which maximise the evidence lower bound.

In the first step we could work on the likelihood function fore each patient to estimate posterior distributions of patient specific variables θ and z. The likelihood function for s^{th} individual is as follows:

$$
\begin{aligned}
&\ln p(\mathbf{w}, \mathbf{z}, \theta|\alpha, \beta) = \ln p(\theta|\alpha) + \sum_{n=1}^{N_s}\{\ln p(z_n|\theta) + \ln p(w_n|z_n, \beta)\}, \\
&p(\theta|\alpha) = \frac{\Gamma(\sum_{i=1}^{K} \alpha_i)}{\prod_i^K \Gamma(\alpha_i)} \prod_{i=1}^{K} \theta_i^{\alpha_i - 1}, \\
&p(z_n|\theta) = \theta_{1(i=z_n)}, \\
&p(w_n|z_n, \beta(t_n)) = \beta_{1(i=z_n),1(j=w_n)}(t_n).
\end{aligned}
\tag{1}
$$

Due to the computational cost of simultaneously modelling hundreds of diseases in the biobank and the inference accuracy consideration (which we will explain in section 4.4), we adopted a collapsed variational methods for this step. The method is motivated by [22]. Detailed explanation on why we chose this rather sophisticated methods rather than the commonly used mean filed methods is discussed in section 4.4, for those interested.

In the second step, we treated the β as parameter of the model and seek to maximise the evidence function $p(\mathbf{w}|\alpha, \beta)$ (obtained by integrate out θ and z from the likelihood function). Directly working on the evidence function is implausible, therefore we work on the evidence lower bound, where we made use of the posterior distribution $q(\mathbf{z}, \theta)$ estimated in previous step.

$$
\mathcal{L}(\mathbf{z}, \theta, \beta, \alpha) = \mathbf{E}_q\{\ln p(\mathbf{w}, \mathbf{z}, \theta|\alpha, \beta) - \ln q(\mathbf{z}, \theta)\}. \tag{2}
$$

We will see this is still not easily achieved, therefore we applied a local variational method to find an approximate solution. For an easy introduction to local variational inference, see chapter 10.5 of [23]. Details of the inference will be explained in section 4.3.

4.3 Inference of model posterior distribution and parameters

The model inference will be performed by alternation an E-step and a M-step. The EM algorithm will guarantee good convergence properties. For both steps, varia-

tional methods will be used to approximate the distribution, though the techniques are very different. We have tested under realistic parameters, these approximated distribution are close to the true distribution.

4.3.1 Collapsed variational inference to estimate patient-level posterior distribution $q(z, \theta)$

In this section, we explained how we found the detailed expression of the lower bound function in equation 2. Details below lay out how we found a variational distribution of $\mathbf{z}$ (equation 8) and use this distribution to compute the evidence lower bound in equation 9 and equation 10. For those who are not interested in the detailed derivation and rationale behind multiple choices of approximation methods, these two equations are all you need to know.

The variational inference aims to approximate posterior $p(\mathbf{z}, \theta|\mathbf{w}, \alpha, \beta)$ using variational distributions $q(\mathbf{z}, \theta)$ that has structure assumptions, which makes them easier to estimate. The most widely used form of variational distribution is the factorised ones, where we assume target posterior distributions are independently distributed, i.e. $q(\mathbf{z}, \theta) = q(\mathbf{z})q(\theta)$. We will derive the inference using this assumption and compare it with the collapsed variational inference in section 4.4.

The latent variable model using Dirichlet distribution is typically designed to model text, where a document is equivalent as a patient in our model. A document will have thousands of words (equivalent of our diagnoses), which provides strong information to fit $q(\mathbf{z}, \theta)$ with strong assumptions. When data has less diagnoses per patient, a variational distributions with less stringent assumptions are preferred, which will increase approximation accuracy. Here we adopted a collapsed variational method, which put less assumptions on the variational distribution and is more accurate than the mean-field variational inference method. [22] The idea is to only assume a factorization over $q(\mathbf{z})$, but not between $\mathbf{z}$ and θ. Therefore the assumptions and lower bound of evidence became (note we are considering likelihood function for only the sth patient from now on, as all of patients are independent conditional on α, β):

$$\begin{aligned} q(\mathbf{z}, \theta) &= q(\theta|\mathbf{z}) \prod_n q(z_n), \\ \mathcal{L}(\mathbf{z}, \theta, \beta, \alpha) &= \mathbf{E}_q\{\ln p(\mathbf{w}, \mathbf{z}, \theta|\alpha, \beta) - \ln q(\mathbf{z}) - \ln q(\theta|\mathbf{z})\} \\ &= \mathbf{E}_{q(z)}\{\mathbf{E}_{q(\theta|z)}\{\ln p(\mathbf{w}, \mathbf{z}, \theta|\alpha, \beta) - \ln q(\theta|\mathbf{z})\} - \ln q(\mathbf{z})\} \end{aligned} \tag{3}$$

Maximise $\mathbf{E}_{q(\theta|z)}\{\ln p(\mathbf{w}, \mathbf{z}, \theta|\alpha, \beta) - \ln q(\theta|\mathbf{z})\}$ with respect to $q(\theta|\mathbf{z})$ will give us $q(\theta|\mathbf{z}) = p(\theta|\mathbf{w}, \mathbf{z}, \alpha, \beta)$. The maximisation is achieved similarly to the mean-field approximation where the evidence is decomposed into a lower bound and KL divergence, where lower bound is maximised when KL divergence is 0. After

minimising with respect to $q(\theta|\mathbf{z})$ [22], the lower bound could be simplified to:

$$\mathcal{L}(\mathbf{z},\theta,\beta,\alpha) = \mathbf{E}_{q(\mathbf{z})}\{\ln p(\mathbf{w},\mathbf{z}|\alpha,\beta) - \ln q(\mathbf{z})\}$$

The optimisation of this lower bound is similar to collapsed Gibbs sampling, where we first marginalise over θ.

$$\begin{aligned} p(\mathbf{w},\mathbf{z}|\alpha,\beta) &= \int_\theta \frac{\Gamma(\sum_{i=1}^K \alpha_i)}{\prod_i^K \Gamma(\alpha_i)} \prod_{i=1}^K \theta_i^{\alpha_i + \sum_n z_{ni} - 1} \cdot \prod_{i=1}^K \prod_{j=1}^D \beta_{ij}^{\sum_n z_{ni} w_{nj}} \\ &= \frac{\Gamma(\sum_{i=1}^K \alpha_i)}{\prod_i^K \Gamma(\alpha_i)} \frac{\prod_i^K \Gamma(\alpha_i + \sum_n z_{ni})}{\Gamma(\sum_{i=1}^K \alpha_i + N_s)} \cdot \prod_{i=1}^K \prod_{j=1}^D \beta_{ij}^{\sum_n z_{ni} w_{nj}}. \end{aligned} \tag{4}$$

From this marginal complete data likelihood, we could derive the conditional distribution $p(z_{n'} = k|\mathbf{z}_{\neg\mathbf{n}'},\mathbf{w},\alpha,\beta)$ (as in collapsed gibbs sampling) to evaluate the dependency within $\mathbf{z}$. Here $\neg n'$ refer to indices of all words excluding n'.

$$\begin{aligned} p(z_{n'}|\mathbf{z}_{\neg\mathbf{n}'},\mathbf{w},\alpha,\beta) &= \frac{p(z_{n'},\mathbf{z}_{\neg\mathbf{n}'},\mathbf{w}|\alpha,\beta)}{p(\mathbf{z}_{\neg\mathbf{n}},\mathbf{w}|\alpha,\beta)} \\ &= \frac{p(\mathbf{z},\mathbf{w}|\alpha,\beta)}{p(\mathbf{z}_{\neg\mathbf{n}}, w_{\neg n}|\alpha,\beta) p(w_{n'}|\alpha,\beta)} \\ &\propto \frac{\prod_i^K \Gamma(\alpha_i + \sum_n z_{ni}) \prod_{i=1}^K \prod_{j=1}^D \beta_{ij}^{\sum_n z_{ni} w_{nj}}}{\prod_i^K \Gamma(\alpha_i + \sum_{\neg n'} z_{ni}) \prod_{i=1}^K \prod_{j=1}^D \beta_{ij}^{\sum_{\neg n'} z_{ni} w_{nj}}} \\ &\propto \prod_i^K (\alpha_i + \sum_{n \in \neg n'} z_{ni})^{z_{n'i}} \prod_{i=1}^K \prod_{j=1}^D \beta_{ij}^{z_{n'i} w_{n'j}}. \end{aligned} \tag{5}$$

For a large N_s, $(\alpha_i + \sum_{n\in\neg n'} z_{ni})$ will be approximately the same across n', therefore $z_{n'}$ will be less dependent on $\mathbf{z}_{\neg n'}$.

$$\lim_{N_s\to\infty} p(z_{n'}|\mathbf{z}_{\neg\mathbf{n}'},\mathbf{w},\alpha,\beta) \propto \prod_i^K \left[(\alpha_i + N_s\theta_i) \prod_{j=1}^D \beta_{ij}^{w_{n'j}}\right]^{z_{n'i}},$$

where distribution of $\mathbf{z}$ factorises over n within a single subjects. Therefore, the $q^*(\mathbf{z})$ in equation 17 which factorises over n (each $q^*(z_{n'})$ is independent of other diagnosis $\mathbf{z}_{\neg n'}$) could approximate $p(\mathbf{z}|\mathbf{w},\alpha,\beta)$ accurately. However, N_s is likely to be small in the patient dataset, therefore the mean-field approximation in equation 17 would be less accurate as it does not include any dependency between $\mathbf{z}_n$ and $\mathbf{z}_{\neg\mathbf{n}'}$. We therefore adopt the strategies proposed by Teh et al to use variatioanal

distribution to approximate the marginal distribuion in equation 4:

$$
\begin{aligned}
\ln q^*(z_{n'}) &= \mathbf{E}_{q(\mathbf{z}_{\neg \mathbf{n'}})}\{\ln p(\mathbf{w}, \mathbf{z}|\alpha, \beta)\} \\
&= \mathbf{E}_{q(\mathbf{z}_{\neg \mathbf{n'}})}\{\sum_{i=1}^{K} \ln \Gamma(\alpha_i + \sum_{n} z_{ni}) + \sum_{i=1}^{K}\sum_{j=1}^{D} z_{n'i} w_{n'j} \ln \beta_{ij}\} + const(z_{n'}) \\
&= \sum_{i=1}^{K} z_{n'i} \Big(\mathbf{E}_{q(\mathbf{z}_{\neg \mathbf{n'}})}\{\ln(\alpha_i + \sum_{n \in \neg n'} z_{ni})\} + \sum_{j=1}^{D} w_{n'j} \ln \beta_{ij} \Big) + const(z_{n'})
\end{aligned} \tag{6}
$$

We now have the form of multinomial distribution of $z_{n'}$. The key lies in how to estimate $\mathbf{E}_{q(\mathbf{z}_{\neg \mathbf{n'}})}\{\ln(\alpha_i + \sum_{n \in \neg n'} z_{ni})\}$. Teh et al [22] proposed a Gaussian approximation which could improve computation efficiency by magnitudes. We first expand $\ln(\alpha_i + \sum_{n \in \neg n'} z_{ni})$ as a function of $\sum_{n \in \neg n'} z_{ni}$ at n_0 by Taylor expansion:

$$
\ln(\alpha_i + \sum_{n \in \neg n'} z_{ni}) = \ln(\alpha_i + n_0) + \frac{\sum_{n \in \neg n'} z_{ni} - n_0}{(\alpha_i + n_0)} - \frac{(\sum_{n \in \neg n'} z_{ni} - n_0)^2}{2(\alpha_i + n_0)^2},
$$

where we included only first two terms. If setting $n_0 = \mathbf{E}_{q(\mathbf{z}_{\neg \mathbf{n'}})}\{\sum_{n \in \neg n'} z_{ni}\} = \sum_{n \in \neg n'} \mathbf{E}_q\{z_{ni}\}$, we get:

$$
\mathbf{E}_{q(\mathbf{z}_{\neg \mathbf{n'}})}\{\ln(\alpha_i + \sum_{n \in \neg n'} z_{ni})\} = \ln(\alpha_i + n_0) - \frac{Var_q[\sum_{n \in \neg n'} z_{ni}]}{2(\alpha_i + n_0)^2}. \tag{7}
$$

where, $Var_q[\sum_{n \in \neg n'} z_{ni}] = \sum_{n \in \neg n'} (1 - \mathbf{E}_q\{z_{ni}\})\mathbf{E}_q\{z_{ni}\}$ .Plugging this into equation 6 and notice the normalization of multinomial distribution, we get:

$$
z_{n'i} \sim \mathbf{Cat}\Big(\frac{(\alpha_i + n_0) \exp\big(- \frac{Var_q[\sum_{n \in \neg n'} z_{ni}]}{2(\alpha_i + n_0)^2} + \sum_{j=1}^{D} w_{n'j} \ln \beta_{ij} \big)}{\sum_{i=1}^{K} (\alpha_i + n_0) \exp\big(- \frac{Var_q[\sum_{n \in \neg n'} z_{ni}]}{2(\alpha_i + n_0)^2} + \sum_{j=1}^{D} w_{n'j} \ln \beta_{ij} \big)} \Big). \tag{8}
$$

For prediction tasks, the posterior θ could be evaluated using distribution of $\mathbf{z}$.

$$
\theta \sim \mathbf{Dir}(\alpha + \sum_{n=1}^{N_s} \mathbf{E}_q\{z_n\})
$$

Once we have the variational distribution of $\mathbf{z}$ in equation 8, we could used the distributio to compute the expectation in the evidence lower bound (the target objective function to maximise).The evidence lower bound over all subjects is as follows:

$$
\begin{aligned}
\mathcal{L}(\mathbf{z},\theta,\beta,\alpha) =& \mathbf{E}_{q(\mathbf{z})}\{\ln p(\mathbf{w},\mathbf{z}|\alpha,\beta) - \ln q(\mathbf{z})\} \\
=& \sum_{s=1}^{M} \Big(\ln\Gamma(\sum_{i=1}^{K}\alpha_i) - \sum_{i}^{K}\ln\Gamma(\alpha_i) - \ln\Gamma(N_s + \sum_{i=1}^{K}\alpha_i) + \\
& \sum_{i=1}^{K}\mathbf{E}_{q(\mathbf{z})}\{\ln\Gamma(\alpha_i + \sum_{n} z_{ni})\} + \\
& \sum_{n=1}^{N_s}\sum_{i=1}^{K}\mathbf{E}\{z_{sni}\}\sum_{j=1}^{D} w_{snj}\ln\beta_{ij}\Big) - \\
& \sum_{s=1}^{M}\Big(\sum_{n=1}^{N_s}\sum_{i=1}^{K}\mathbf{E}\{z_{ni}\}\ln\mathbf{E}\{z_{ni}\}\Big),
\end{aligned} \tag{9}
$$

where we need to approximate $\mathbf{E}_{q(\mathbf{z})}\{\ln\Gamma(\alpha_i + \sum_n z_{ni})\}$. Making use of the Stirling's approximation, we found $\ln\Gamma(z) = (z - \frac{1}{2})\ln(z) - z + \frac{1}{12z} + \frac{1}{2}\ln(2\pi)$ could approximate $\ln\Gamma(z)$ accurately for $z > 1$. Therefore, by plugging in Stirling's approximation and reuse equation 7 we could approximate this expectation:

$$
\begin{aligned}
\mathbf{E}_{q(\mathbf{z})}\{\ln\Gamma(\alpha_i + \sum_n z_{ni})\} =& \mathbf{E}_{q(\mathbf{z})}\{(\alpha_i + \sum_n z_{ni})\ln(\alpha_i + \sum_n z_{ni}) \\
& - \frac{1}{2}\ln(\alpha_i + \sum_n z_{ni}) - (\alpha_i + \sum_n z_{ni}) + \frac{1}{12(\alpha_i + \sum_n z_{ni})} + \frac{1}{2}\ln(2\pi)\} \\
=& \mathbf{E}_{q(\mathbf{z})}\{(\alpha_i + n_0)\ln(\alpha_i + n_0) + \frac{(\sum_n z_{ni} - n_0])^2}{2(\alpha_i + n_0)} \\
& - \frac{1}{2}\ln(\alpha_i + n_0) + \frac{(\sum_n z_{ni} - n_0])^2}{4(\alpha_i + n_0)2} \\
& - (\alpha_i + \sum_n z_{ni}) \\
& + \frac{1}{12(\alpha_i + n_0)} + \frac{(\sum_n z_{ni} - n_0])^2}{12(\alpha_i + n_0)^3} + \frac{1}{2}\ln(2\pi)\} \\
=& (\alpha_i + n_0)\ln(\alpha_i + n_0) - \frac{1}{2}\ln(\alpha_i + n_0) - (\alpha_i + n_0) + \frac{1}{12(\alpha_i + n_0)} \\
& + Var_q[\sum_n z_{ni}]\big(\frac{1}{2(\alpha_i + n_0)} + \frac{1}{4(\alpha_i + n_0)2} + \frac{1}{12(\alpha_i + n_0)^3}\big) \\
& + \frac{1}{2}\ln(2\pi),
\end{aligned} \tag{10}
$$

Note here the first order terms in Taylor expansion are cancelled after taking the expectation and setting $n_0 = \sum_n \mathbf{E}_q\{z_{sni}\}$. The variance are computed by applying the independent assumption over $q(z_n)$: $Var_q[\sum_n z_{ni}] = \sum_n(1 - \mathbf{E}_q\{z_{ni}\})\mathbf{E}_q\{z_{ni}\}$.

4.3.2 Estimate topic profiles $\beta(t)$

In the simple topic models[24], the topic values could be estimated by directly maximising the evidence lower bound with a constraint $\sum_{j=1}^{D} \beta_{ij} = 1$, which is described in section 4.4 for completeness and comparison. Here we estimate the topic as functions of age by parameterising each β_{ij} as a function of age. The only related term in likelihood function (equation 1) is:

$$\ln p(w_n|z_n, \beta(t_n)) = \sum_{i=1}^{K} z_{sni} \sum_{j=1}^{D} w_{snj} \ln \pi(\beta_{ij}(t_n)),$$

where we use softmax function to ensure topics are multinomial distributions:

$$\pi(\beta_{ij}(t_n)) = \frac{\exp(\boldsymbol{\beta}_{ij}^T \phi(t_n))}{\sum_{j=1}^{D} \exp(\boldsymbol{\beta}_{ij}^T \phi(t_n))}.$$

We used spline/polynomial functions to model age. The goal is to estimate spline/polynomial coefficients $\boldsymbol{\beta}_{ij} = \{\beta_{ijd}\}$, $d = 1, 2, ..., P$, where P is the degree of freedom that controls the smoothness. $\phi(t_n)$ is polynomial or spline basis. Notice here the scale of $\boldsymbol{\beta}_{ij} = \{\beta_{ijd}\}$ does not matter, as we could subtract same intercept from the exponential in both numerator and denominator to change in the scale. However, in practice we put a prior $\mathcal{N}(\boldsymbol{\beta}_{ij}|0, \sigma_0^2\mathbf{I})$ on β_{ij} to regularise the search space of the gradient descent optimization described below. Here we choose a non-informative prior with large variance, $\sigma_0^2 = 100$.

To maximise the evidence lower bound, we notice that $\ln(\cdot)$ is a concave function and by Taylor expansion:

$$\ln(\sum_{j=1}^{D} \exp(\boldsymbol{\beta}_{ij}^T \phi(t_{sn}))) \leq \ln \zeta + \zeta^{-1}(\sum_{j=1}^{D} \exp(\boldsymbol{\beta}_{ij}^T \phi(t_{sn})) - \zeta).$$

Therefore, by introducing a variational variable ζ, we find following lower bound

of the ELBO function $\mathcal{L}$ with respect to $\boldsymbol{\beta}_{ij}$:

$$\begin{aligned}\mathcal{L}_{[\beta]} &= \sum_{s=1}^{M}\sum_{n=1}^{N_s} \mathbf{E}_q\{\ln p(w_n|z_n, \beta(t_{sn}))\} \\ &= \sum_{s=1}^{M}\sum_{n=1}^{N_s}\sum_{i=1}^{K}\sum_{j=1}^{D} \Big(\boldsymbol{\beta}_{ij}^T\phi(t_{sn}) - \ln(\sum_{j'=1}^{D}\exp\{\boldsymbol{\beta}_{ij'}^T\phi(t_{sn})\})\Big)\mathbf{E}\{z_{sni}\}w_{snj} \geq \\ &\sum_{s=1}^{M}\sum_{n=1}^{N_s}\sum_{i=1}^{K}\sum_{j=1}^{D} \big(\boldsymbol{\beta}_{ij}^T\phi(t_{sn}) - \zeta_{sni}^{-1}\sum_{j'=1}^{D}\exp\{\boldsymbol{\beta}_{ij'}^T\phi(t_{sn})\} - \ln\zeta_{sni} + 1\big)\mathbf{E}\{z_{sni}\}w_{snj}.\end{aligned} \tag{11}$$

We could then apply a method called local variational inference to maximise the right hand side of equation 11. We do this by updating $\boldsymbol{\beta}$ and ζ in turn. Take derivative with respect to ζ_{sni}, we obtained following update:

$$\zeta_{sni} = \sum_{j=1}^{D}\exp\{\boldsymbol{\beta}_{ij}^T\phi(t_{sn})\} \tag{12}$$

In order to update the lower bound with respect to $\boldsymbol{\beta}$, we separate the terms containing $\boldsymbol{\beta}_{ij}$:

$$\mathcal{L}_{[\beta_{ij}]} = \sum_{s=1}^{M}\sum_{n=1}^{N_s}\mathbf{E}\{z_{sni}\}w_{snj}\boldsymbol{\beta}_{ij}^T\phi(t_{sn}) - \sum_{s=1}^{M}\sum_{n=1}^{N_s}\mathbf{E}\{z_{sni}\}\zeta_{sni}^{-1}\exp\{\boldsymbol{\beta}_{ij}^T\phi(t_{sn})\}.$$

There is no analytical solution for $\boldsymbol{\beta}_{ij}$, but the lower bound is convex so we could maximize the lower bound using following gradient information:

$$\nabla_{\boldsymbol{\beta}_{ij}}\mathcal{L}_{[\beta]} = \sum_{s=1}^{M}\sum_{n=1}^{N_s}\Big(\mathbf{E}\{z_{sni}\}w_{snj} - \mathbf{E}\{z_{sni}\}\zeta_{sni}^{-1}\exp\{\boldsymbol{\beta}_{ij}^T\phi(t_{sn})\}\Big)\phi(t_{sn}) \tag{13}$$

The gradient information of $\mathcal{L}_{[\beta_{ij}]}$ allows efficient numeric estimation of $\boldsymbol{\beta}_{ij}$. However, evaluating $\mathcal{L}_{[\beta_{ij}]}$ and $\nabla_{\boldsymbol{\beta}_{ij}}\mathcal{L}_{[\beta]}$ is computational expensive due to $\exp\{\boldsymbol{\beta}_{ij}^T\phi(t_{sn})\}$, which require looping through s, n (all diagnosis records across all subjects!). The gradient descent methods for estimating $\boldsymbol{\beta}_{ij}$ requires evaluating$\mathcal{L}_{[\beta_{ij}]}$ and $\nabla_{\boldsymbol{\beta}_{ij}}\mathcal{L}_{[\beta]}$ at each gradient step, which prohibit scaling up the model to large data set. To solve this problem in practice we discretise t_{sn} into years which allows us to pre-compute the sum of $\mathbf{E}\{z_{sni}\}\zeta_{sni}^{-1}$ over all incidences that happened at each age year. For each new $\boldsymbol{\beta}_{ij}$, we could then sum over years instead of across all diagnoses, which reuses the sums computed for each year. This trick significant reduced the computation cost of evaluating $\mathcal{L}_{[\beta_{ij}]}$ and $\nabla_{\boldsymbol{\beta}_{ij}}\mathcal{L}_{[\beta]}$ which makes the estimation of

age topics over the entire UK Biobank HES possible (order of growth is multiplied by $O(\frac{1}{\sum_{s=1}^{M} N_s})$, where $\sum_{s=1}^{M} N_s$ is the total number of diagnoses, which is 1,726,144 for UK Biobank). In conclusion, we could update $\boldsymbol{\beta}$ using following psuedo-code:

Algorithm 1: Maximize local variationl lower bound

```
initialization;
for i ← 1 to K do
    for j ← 1 to D do
        Update ζ_sni = Σ_{j=1}^{D} exp{β_ij^T φ(t_sn)} ;
        Update β_ij to maximize L_[β_ij] ;
    end
end
```

Note here we need to update ζ_{sni} for each j, while in practice we only update ζ_{sni} once for each optimization of $\boldsymbol{\beta}$ to allow parallel computation over j.

The above computation provides a point estimate for $\boldsymbol{\beta}$, which we adopted when applying our methods to empirical data. We also provide the mathematical derivation for posterior distributions of $\boldsymbol{\beta}$, though we do not present results on empirical data and the full Bayesian method is **not** implemented in ATM software, due to computational cost. We used a Gaussian prior for $\beta_{ijd} \sim \mathcal{N}(0, \sigma^2)$. A full variational inference of β is performed by maximising following evidence lower bound:

$$\begin{aligned}
\mathcal{L}_{[\beta]} &= \sum_{s=1}^{M}\sum_{n=1}^{N_s} \mathbf{E}_q\{\ln p(w_n|z_n, \beta(t_{sn}))\} + \sum_{i=1}^{K}\sum_{j=1}^{D}\sum_{d=1}^{P} \Big(\mathbf{E}_q\{\ln p(\boldsymbol{\beta}_{ijd})\} - \mathbf{E}_q\{\ln q(\boldsymbol{\beta}_{ijd})\}\Big) \\
&= \sum_{s=1}^{M}\sum_{n=1}^{N_s}\sum_{i=1}^{K}\sum_{j=1}^{D} \big(\mathbf{E}_q\{\boldsymbol{\beta}_{ij}\}^T\phi(t_{sn}) - \mathbf{E}_q\{\ln(\sum_{j=1}^{D}\exp(\boldsymbol{\beta}_{ij}^T\phi(t_{sn})))\}\big)\mathbf{E}\{z_{sni}\}w_{snj} - \\
&\frac{1}{2\sigma^2}\sum_{i=1}^{K}\sum_{j=1}^{D}\sum_{d=1}^{P}\mathbf{E}_q\{\boldsymbol{\beta}_{ijd}^2\} - \sum_{i=1}^{K}\sum_{j=1}^{D}\sum_{d=1}^{P}\mathbf{E}_q\{\ln q(\boldsymbol{\beta}_{ijd})\} \geq \\
&\sum_{s=1}^{M}\sum_{n=1}^{N_s}\sum_{i=1}^{K}\sum_{j=1}^{D} \big(\mathbf{E}_q\{\boldsymbol{\beta}_{ij}\}^T\phi(t_{sn}) - \zeta_{sni}^{-1}\sum_{j=1}^{D}\mathbf{E}_q\{\exp(\boldsymbol{\beta}_{ij}^T\phi(t_{sn}))\} - \ln\zeta_{sni} + 1\big)\mathbf{E}\{z_{sni}\}w_{snj} - \\
&\frac{1}{2\sigma^2}\sum_{i=1}^{K}\sum_{j=1}^{D}\sum_{d=1}^{P}\mathbf{E}_q\{\boldsymbol{\beta}_{ijd}^2\} - \sum_{i=1}^{K}\sum_{j=1}^{D}\sum_{d=1}^{P}\mathbf{E}_q\{\ln q(\boldsymbol{\beta}_{ijd})\}.
\end{aligned} \tag{14}$$

Following [25], we assumed an independent variational Gaussian distribution for each β_{ijd}:

$$q(\beta_{ijd}) = \mathcal{N}(\beta_{ijd}|\lambda_{ijd}, \nu_{ijd}^2),$$

and observe the moment-generating function of Guanssian distribution is:

$$\mathbf{E}_q\{\exp(\boldsymbol{\beta}_{ijd}\phi_d(t_{sn}))\} = \exp\Big(\phi_d(t_{sn})\lambda_{ijd} + \frac{\phi_d^2(t_{sn})\nu_{ijd}^2}{2}\Big),$$

we obtain a tractable lower bound with respect to the variational parameters $\{\zeta_{sni}, \lambda_{ijd}, \nu_{ijd}^2\}$:

$$\mathcal{L}_{\zeta,\lambda,\nu^2} = \sum_{s=1}^{M}\sum_{n=1}^{N_s}\sum_{i=1}^{K}\sum_{j=1}^{D}\big(\lambda_{ij}^T\phi(t_{sn}) - \zeta_{sni}^{-1}\sum_{j=1}^{D}\exp\{\sum_{d=1}^{P}\Big(\phi_d(t_{sn})\lambda_{ijd} + \frac{\phi_d^2(t_{sn})\nu_{ijd}^2}{2}\Big)\} - \ln\zeta_{sni} + 1\big)\cdot\mathbf{E}\{z_{sni}\}w_{snj} - \sum_{i=1}^{K}\sum_{j=1}^{D}\sum_{d=1}^{P}\big(\frac{1}{2\sigma^2}\nu_{ijd}^2 + \frac{1}{2}\ln\nu_{ijd}^2\big). \tag{15}$$

Iteratively maximising above evidence lower bound with respect to $\{\zeta_{sni}, \lambda_{ijd}, \nu_{ijd}^2\}$ estimates posterior distribution $q(\beta_{ijd})$ which provides uncertainty quantification of β_{ijd}.

4.4 Comparison of collapsed variational inference and mean field variational inference

A vast number of inference methods have been developed for models based on original Latent Dirichlet Allocation. The most prominent of which are collapsed Gibbs sampling and mean field variational inference. For inference of model with exchangeable variables using extremely large and noisy data set, it is desirable to have a deterministic method such as variational inference. Collapsed variational inference makes less assumptions for approximation, therefore the inferred distributions are strictly closer to the true posterior distributions than the mean-field variational Bayesian methods. We will explain why accuracy is important for the diagnosis data in section 4.4.2.

4.4.1 Mean field variational inference to estimate patient-level posterior distribution $q(z, \theta)$

Please note this section is just a replication of [24] using our notation, which is provided to make the note self-contained. We assume that variational distributions for latent variables θ and $\mathbf{z}$ are independent of each other, then we get the variational lower bound for the log likelihood of a single subject:

$$\begin{aligned} &q(\mathbf{z},\theta) = q(\mathbf{z})q(\theta), \\ &\mathcal{L}(\mathbf{z},\theta,\beta,\alpha) = \mathbf{E}_q\{\ln p(\mathbf{w},\mathbf{z},\theta|\alpha,\beta) - \ln q(\mathbf{z},\theta)\}. \end{aligned} \tag{16}$$

It is straightforward to estimate $q(\mathbf{z})$ and $q(\theta)$ that maximise the lower bound $\mathcal{L}(\mathbf{z}, \theta, \beta, \alpha)$:

$$\begin{aligned}
\ln q^*(\theta) &= \ln p(\theta|\alpha) + \mathbf{E}_{q(\mathbf{z})}\{\sum_{n=1}^{N_s} \ln p(z_n|\theta)\} + const \\
&= \sum_{i=1}^{K}(\alpha_i + \sum_{n=1}^{N_s} \mathbf{E}\{z_{ni}\} - 1) \ln \theta_i + const, \\
\ln q^*(\mathbf{z}) &= \mathbf{E}_{q(\theta)}\{\sum_{n=1}^{N_s} \ln p(z_n|\theta)\} + \sum_{n=1}^{N_s} \ln p(w_n|z_n, \beta) + const \\
&= \sum_{n=1}^{N_s}\sum_{i=1}^{K} z_{ni}\Big(\mathbf{E}\{\ln \theta_i\} + \sum_{j=1}^{D} w_{nj} \ln \beta_{ij}\Big) + const.
\end{aligned} \tag{17}$$

We see that $q(\theta)$ factorises over i and $q(\mathbf{z})$ factorises over n, i. Therefore, we get the variational distribution for $\mathbf{z}$ and θ:

$$\theta_i \sim \mathbf{Dir}(\alpha_i + \sum_{n=1}^{N_s} \mathbf{E}\{z_{ni}\})$$

$$z_{ni} \sim \mathbf{Cat}\Big(\frac{\exp\big(\mathbf{E}\{\ln \theta_i\} + \sum_{j=1}^{D} w_{nj} \ln \beta_{ij}\big)}{\sum_{i=1}^{K} \exp\big(\mathbf{E}\{\ln \theta_i\} + \sum_{j=1}^{D} w_{nj} \ln \beta_{ij}\big)}\Big)$$

We then has the $(m+1)^{th}$ E-step as follows:

$$\begin{aligned}
\mathbf{E}^{m+1}\{\ln \theta_i\} &= \Psi(\alpha_i + \sum_{n=1}^{N_s} \mathbf{E}^m\{z_{ni}\}) - \Psi(\sum_{i=1}^{K} \big(\alpha_i + \sum_{n=1}^{N_s} \mathbf{E}^m\{z_{ni}\}\big)), \\
\mathbf{E}^{m+1}\{z_{ni}\} &= \frac{\exp\big(\mathbf{E}^{m+1}\{\ln \theta_i\} + \sum_{j=1}^{D} w_{nj} \ln \beta_{ij}^m\big)}{\sum_{i=1}^{K} \exp\big(\mathbf{E}^{m+1}\{\ln \theta_i\} + \sum_{j=1}^{D} w_{nj} \ln \beta_{ij}^m\big)},
\end{aligned} \tag{18}$$

where $\mathbf{E}^m$ and β^m refers to the estimation of previous step (m^{th} step); Ψ is the digamma function.

To perform the M-step, we maximize the lower bound $\mathcal{L}$ in equation 2 for the

entire population.

$$
\begin{aligned}
\mathcal{L}(\mathbf{z},\theta,\beta,\alpha) = \sum_{s=1}^{M} \Big(& \ln\Gamma(\sum_{i=1}^{K}\alpha_i) - \sum_{i}^{K}\ln\Gamma(\alpha_i) + \sum_{i=1}^{K}(\alpha_i - 1)\mathbf{E}\{\ln\theta_{si}\} + \\
& \sum_{n=1}^{N_s}\sum_{i=1}^{K}(\mathbf{E}\{z_{sni}\}\mathbf{E}\{\ln\theta_{si}\}) + \\
& \sum_{n=1}^{N_s}\sum_{i=1}^{K}\mathbf{E}\{z_{sni}\}\sum_{j=1}^{D} w_{snj}\ln\beta_{ij}\Big) - \\
& \sum_{s=1}^{M}\Big(\ln\Gamma(\sum_{i=1}^{K}(\alpha_i + \sum_{n=1}^{N_s}\mathbf{E}\{z_{ni}\})) - \sum_{i=1}^{K}\ln\Gamma(\alpha_i + \sum_{n=1}^{N_s}\mathbf{E}\{z_{ni}\}) + \\
& \sum_{i=1}^{K}(\alpha_i + \sum_{n=1}^{N_s}\mathbf{E}\{z_{ni}\} - 1)\mathbf{E}\{\ln\theta_{si}\} + \\
& \sum_{n=1}^{N_s}\sum_{i=1}^{K}\mathbf{E}\{z_{ni}\}\ln\mathbf{E}\{z_{ni}\}\Big)
\end{aligned} \tag{19}
$$

For β, we take terms in $\mathcal{L}$ and add Lagrange multipliers:

$$
\mathcal{L}_{[\beta]} = \sum_{i=1}^{K}\sum_{j=1}^{D}\ln\beta_{ij}\sum_{s=1}^{M}\sum_{n=1}^{N_s}\mathbf{E}\{z_{sni}\}w_{snj} + \sum_{i=1}^{K}\lambda_i(\sum_{j=1}^{D}\beta_{ij} - 1).
$$

Set the derivative of $\mathcal{L}_{[\beta]}$ with respect β to zero, we could get the $(n+1)^{th}$ update for *beta*:

$$
\beta_{ij}^{n+1} = \frac{\sum_{s=1}^{M}\sum_{n=1}^{N_s}\mathbf{E}^{n+1}\{z_{sni}\}w_{snj}}{\sum_{j=1}^{D}\sum_{s=1}^{M}\sum_{n=1}^{N_s}\mathbf{E}^{n+1}\{z_{sni}\}w_{snj}}
$$

The terms in lower bound that contains α are:

$$
\mathcal{L}_{[\alpha_i]} = \sum_{s=1}^{M}\Big(\ln\Gamma(\sum_{i=1}^{K}\alpha_i) - \ln\Gamma(\alpha_i) + \sum_{i=1}^{K}(\alpha_i - 1)\mathbf{E}^{n+1}\{\ln\theta_{si}\}\Big).
$$

Take the derivatives with respect to α:

$$
\frac{\partial\mathcal{L}_{[\alpha]}}{\partial\alpha_i} = M\cdot\Big(\Psi(\sum_{i=1}^{K}\alpha_i) - \Psi(\alpha_i)\Big) + \sum_{s=1}^{M}\mathbf{E}^{n+1}\{\ln\theta_{si}\}.
$$

And the Hessian:

$$
\nabla_{\alpha}^{2}\mathcal{L}_{[\alpha]} = M\cdot\mathrm{diag}(-\Psi^1(\alpha_i)\Big) + M\cdot\Psi^1(\sum_{i=1}^{K}\alpha_i),
$$

where Ψ^1 is the Trigamma function. We use the Newton-Raphson method the find the maximal of α as described in [24]. In practice, we used $\alpha = 1$ to put an uninformative prior robust optimization.

4.4.2 Patients with a few diseases versus documents with many words

In section 4.3.1, we briefly explained why we chose to use collapsed variational inference over a simpler mean-filed variational inference method. We will focus on the difference between equation 17 and equation 5. For the mean-field variational distribution:

$$\ln q^*(\mathbf{z}) = \sum_{n=1}^{N_s}\sum_{i=1}^{K} z_{ni}\Big(\mathbf{E}\{\ln\theta_i\} + \sum_{j=1}^{D} w_{nj}\ln\beta_{ij}\Big) + const,$$

which factorised over the N_s diagnoses. Therefore, the inferred distribution for each z_n is conditional i.i.d.

$$q(z_{n'}|\mathbf{z_{\neg n'}}, \mathbf{w}, \alpha, \beta, \theta) = q(z_{n'}|\mathbf{w}, \alpha, \beta, \theta),$$

Here $\neg n'$ refer to indices of all diagnoses excluding n'. However, for collapsed VB, conditional distribution depends on other diagnoses of the same patient:

$$q(z_{n'}|\mathbf{z_{\neg n'}}, \mathbf{w}, \alpha, \beta) \propto \prod_{i}^{K}(\alpha_i + \sum_{n\in\neg n'} z_{ni})^{z_{n'i}} \prod_{i=1}^{K}\prod_{j=1}^{D}\beta_{ij}^{z_{n'i}w_{n'j}}$$

The impact of the the dependency on the accuracy of posterior approximation depends on the data structure. Most of topic models were designed for text modelling, where each document have a large word number N_s. In this case, $(\alpha_i + \sum_{n\in\neg n'} z_{ni})$ will be approximately the same across n':

$$\lim_{N_s\to\infty} p(z_{n'}|\mathbf{z_{\neg n'}}, \mathbf{w}, \alpha, \beta) \propto \prod_{i}^{K}\Big[(\alpha_i + N_s\theta_i)\prod_{j=1}^{D}\beta_{ij}^{w_{n'j}}\Big]^{z_{n'i}},$$

where θ_i is the topic weight for the sth document. We see $\mathbf{z_{\neg n'}}$ no longer exists and $q^*(\mathbf{z})$ in equation 17 could approximate $p(\mathbf{z}|\mathbf{w}, \alpha, \beta)$ accurately. However, each patient have an average 6.1 distinct diagnoses in UK Biobank HES data, making N_s small for the mean-field approximation. Note, we do not need to assume independence of z_n across diagnoses, it is a consequence of assuming independence between $q(\theta)$ and $q(\mathbf{z})$, which is called induced factorisation in some cases (section 10.2.5 in [23]). In this case collapsed VB models the dependency between $\mathbf{z}_n$ and $\mathbf{z_{\neg n'}}$ and is more accurate at approximating posterior distribution.

References

[22] Teh, Y. W., Newman, D. & Welling, M. A collapsed variational bayesian inference algorithm for latent dirichlet allocation. Tech. Rep., CALIFORNIA UNIV IRVINE SCHOOL OF INFORMATION AND COMPUTER SCIENCE (2007).

[23] Bishop, C. M. & Nasrabadi, N. M. *Pattern recognition and machine learning* (Springer, 2006).

[24] Blei, D. M., Ng, A. Y. & Jordan, M. I. Latent dirichlet allocation. *Journal of machine Learning research* **3**, 993–1022 (2003).

[25] Blei, D. M., Lafferty, J. D. *et al.* A correlated topic model of science. *The annals of applied statistics* **1**, 17–35 (2007).

Supplementary Table Captions

Supplementary Table 1. List of metrics for evaluating ATM performance. The name, purpose, and implementation details of each metric are listed for comparison. For more details of each metric, see Methods.

Supplementary Table 2. Simulation results for ATM on identifying disease subtypes. We show the area under the precision-recall curve (AUPRC) for ATM in simulated data with two subtypes that have 20/10/5 years of age at diagnosis differences. Results for LDA (fifth column) are also shown for comparison. Rows show results for varying proportions of samples that belong to the smaller subtype. The results correspond to Fig. 2.

Supplementary Table 3. Characteristics of disease topics inferred from the UK Biobank. For each topic, we listed the top 10 representative diseases (by topic loadings), heritability estimates, average topic weights (across all individuals), average age (weighted across all disease diagnosis assigned to the topic), proportional of variance explained by BMI, sex, Townsend deprivation index, and birth year.

Supplementary Table 4. Topic loadings of 10 inferred disease topics across 348 diseases in the UK Biobank. For each disease we reported the topic loading across diagnoses before 60 years old and after 60 years old. The Phecode, number of incidences, ICD-10 code, disease name, and Phecode systems are also listed for each disease. The values in this table correspond to Fig. 3.

Supplementary Table 5. Topic loadings as functions of age for 10 inferred disease topics. For each topic, we listed the topic loading of each disease from age 30 to 80 years old. At each age point, the topic loadings add to one across diseases for each topic. The values correspond to Fig. 4A and Supplementary Fig. 9.

Supplementary Table 6. Topic loadings as functions of age for 13 inferred disease topics from the All of Us data. For each topic, we listed the topic loading of each disease between age 20 to 85. At each age point, the topic loadings add to one across diseases for each topic.

Supplementary Table 7. Correlation of topic loadings between each pair of All of Us and UK Biobank topics. Numeric values for Fig. 5B.

Supplementary Table 8. Prevalence in All of Us and UK Biobank for the 233 diseases that are shared between the two data sets.

Supplementary Table 9. Correlations between topic assignments for pairs of All of Us disease and UK Biobank disease across 233 diseases that are shared between the two

data sets. Disease associations to topics are measured using average topic assignments (see Methods for definition) for both UK Biobank and All of Us. Average topic assignments in All of Us are mapped to UK Biobank using topic loading correlation; the correlation for each disease pair in the UK Biobank topic space (Methods).

Supplementary Table 10. List of 52 diseases with comorbidity subtypes in UK Biobank. For each disease with at least 500 diagnoses assigned to each of two discrete subtypes, we list its Phecode, disease description, subtypes with at least 500 diagnoses, and correlations between UK Biobank topic assignments and All of Us topic assignments that were mapped to UK Biobank topics (Methods).

Supplementary Table 11. Number of diagnoses assigned to each subtypes for 52 diseases. We listed the number of diagnoses assigned to each disease subtypes by the diagnosis-specific topic probability, for the 52 diseases that have at least two subtypes with >500 diagnosis.

Supplementary Table 12. Average age at diagnosis for each subtypes of the 52 diseases. We listed the age at diagnosis across all diagnoses within each disease subtype, for the 52 diseases that have at least two subtypes with >500 diagnosis.

Supplementary Table 13. Excess PRS in cases for all topics across 10 diseases (selected by heritability z-score). We report the estimated changes in s.d. of PRS per unit changes in the patient topic weight, which is estimated through regression across disease diagnoses. The PRS was estimated using BOLT-LMM and all the cases of British Isle Ancestry. P-values are for testing association between PRS and patient topic weight. Numbers correspond to Fig. 6B and Extended Data Fig. 6.

Supplementary Table 14. Excess genetic correlations. Columns are: Phecode of the first disease (trait1), Phecode of the second trait (trait2), subtype of the first trait (topic1), subtype of the second trait (topic2), the z-score of genetic correlation between two disease subtypes (subtype.rho.zscore), estimate of genetic correlation between two disease subtypes (subtype.rho.est), standard error of genetic correlation between two disease subtypes (subtyp.rho.err), the z-score of genetic correlation between two diseases (all.rho.zscore), estimate of genetic correlation between two diseases (all.rho.est), standard error of genetic correlation between two diseases (all.rho.err), z-score of excess genetic correlation (diff.zscore), estimate of excess genetic correlation (diff.rg), absolute value of excess genetic correlation (diff.rg.zscore.abs), p-values for z-score test of excess genetic correlation (P), FDR for excess genetic correlation (FDR), name of the first disease (phenotype.1), and name of the second disease (phenotype.2). We only reported p-values of excess genetic correlation when both genetic correlation estimation has standard error <0.1 and at least one of the genetic correlation has |z-score|>4. Numbers correspond to Fig. 7 and Supplementary Fig. 19.

Supplementary Table 15. Heritability estimation for disease subtypes. For each disease we list heritability estimates for two subtypes using LDSC. Topic.x refer to the subtype with the highest heritability, topic.y refer to subtype with the lowest heritability. We report the point estimate, standard error, z-scores of both disease subtypes. The z-score of heritability differences between the two subtypes are also reported. Note we used a different sample threshold 1000 (due to the power of LDSC), which includes 26 of the 52 diseases that have subtypes.

Supplementary Table 16. Excess F_{ST} across disease subtypes. We report the estimate of excess F_{ST} (computed as the F_{ST} across subtypes subtracted by the F_{ST} from controls with matched topic weights). The p-values are for permutation tests of excess $F_{ST}>0$, which is computed from 1000 randomly sampled control sets.

Supplementary Table 17. GxTopic interaction tests across independent GWAS SNPs. Each row represents one SNP x topic weight pair (disease subtype). OR, SE, STAT, and P represent the odds ratio, standard error, test statistics, and p-value of the main effects. SNPxTopic OR, SNPxTopic STAT, SNPxTopic P, SNPxTopic FDR represent the odds ratio, test statistics, p-value for testing interaction regression coefficients, and genome-wide FDR of testing the interaction effect in model 2 of Supplementary Fig. 21. We use study-wise FDR which adjusts for multiple testing across GWAS SNPs of all disease subtypes.

Supplementary Table 18. Significant SNP x topic interactions. Same table as Supplementary Table 17, but filtered to SNP-topic pairs with interaction effect passing FDR<0.1. Reported SNP-topic pairs were selected for topic weights specific effect estimation in Extended Data Fig. 8 and Supplementary Fig. 23.

Supplementary Table 19. Effect size estimation across topic weight quartiles for significant SNP x topic interactions. Quartile, mean_effect, se_effect, refer to the quartile of topic weight, estimate of effect sizes of the SNP using case-controls from this quartile, and standard error of the effect size estimation. We also reported the nearest genes reported by GWAS Catalog. The last two columns report the P-value of effect size being different between the top and bottom quartiles and the FDR (across 2530 tests), indicated by two-sided t-tests.

Supplementary Table 20. Literature search of disease subtypes identified by ATM. We searched on Pubmed using the description (ignoring conjunctions) AND "subtype" in title/abstract and manually screened the top 10 relevant results between 2012 and 2022. The studies that mentioned subtypes of the searched diseases are included in the "Published references" column. If there is no reference of target disease subtypes among the top 10 search results, we use "NA". We note our search is not exhaustive but nevertheless provides information on whether subtypes of the target disease are described in studies involving target disease and subtypes.

Supplementary Table 21. ATM running time. We tested the running time on the UK Biobank data using ATM of varying topic number and parametric form of topic loadings. Degrees of freedom from 2 to 7 represent linear, quadratic polynomial, cubic polynomial, spline with one knot, spline with two knots, and spline with three knots. Note a few models with 50 topics did not converge.

Supplementary Figures

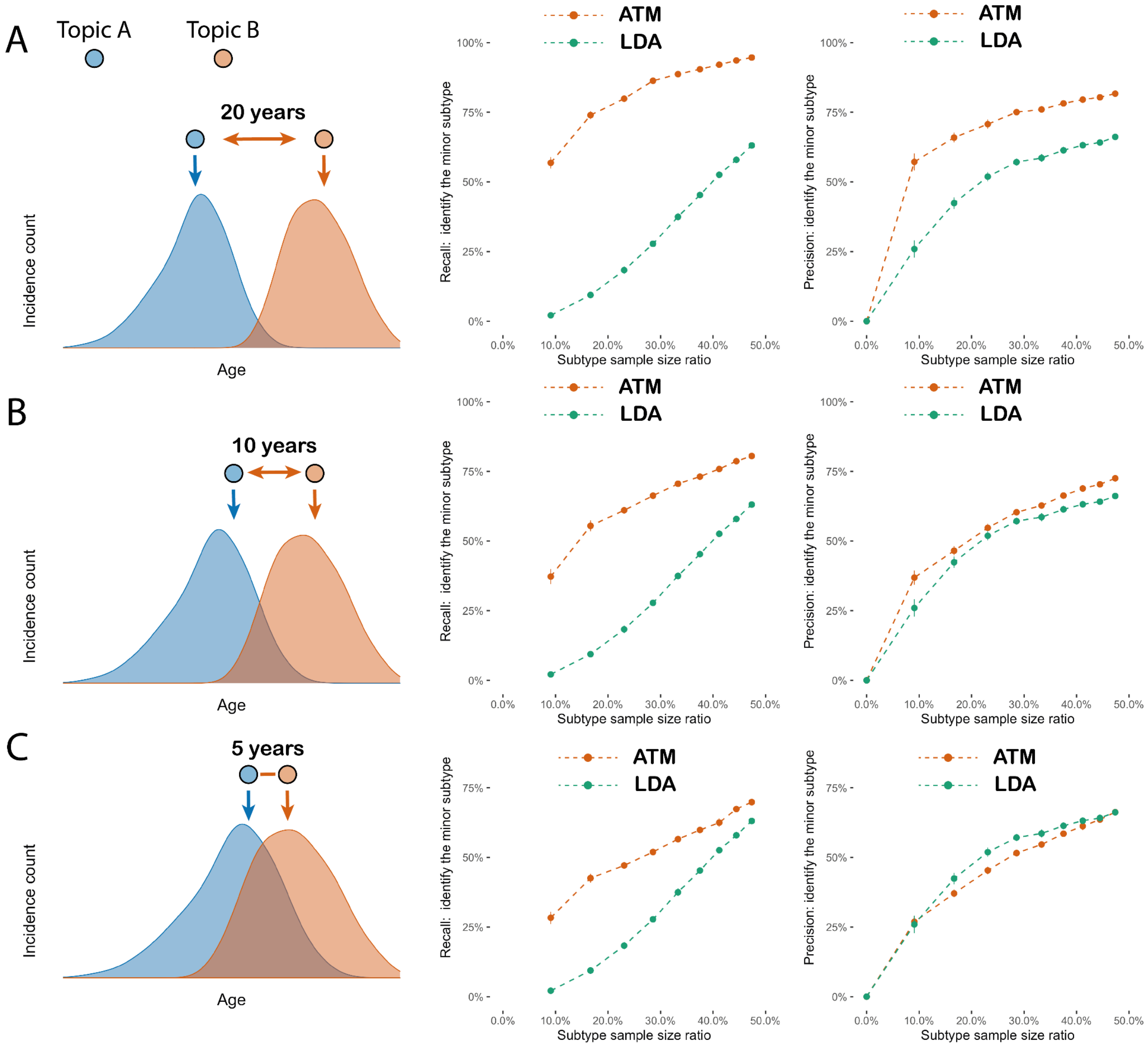

Supplementary Fig. 1. Additional simulation studies established the power of the method to identify comorbidity. The precision and recall rate to correctly assign incident disease to correct comorbidity profiles using Latent Dirichlet Allocation (LDA) and our method (ATM). X-axis refers to the proportion of cases that belong to the small subgroup; precision and recall are computed for the label incidences in the small subgroup. Each dot represents the mean of 100 simulations of 10,000 people, the bar shows the 95% confidence intervals. Red refers to the ATM and green refers to the LDA model.(a) Scenario where two subtypes are simulated with 20 years of difference in age at diagnosis. (b) Scenario where two subtypes are simulated with 10 years of difference in age at diagnosis. (c) Scenario where

two subtypes are simulated with 5 years of age difference.

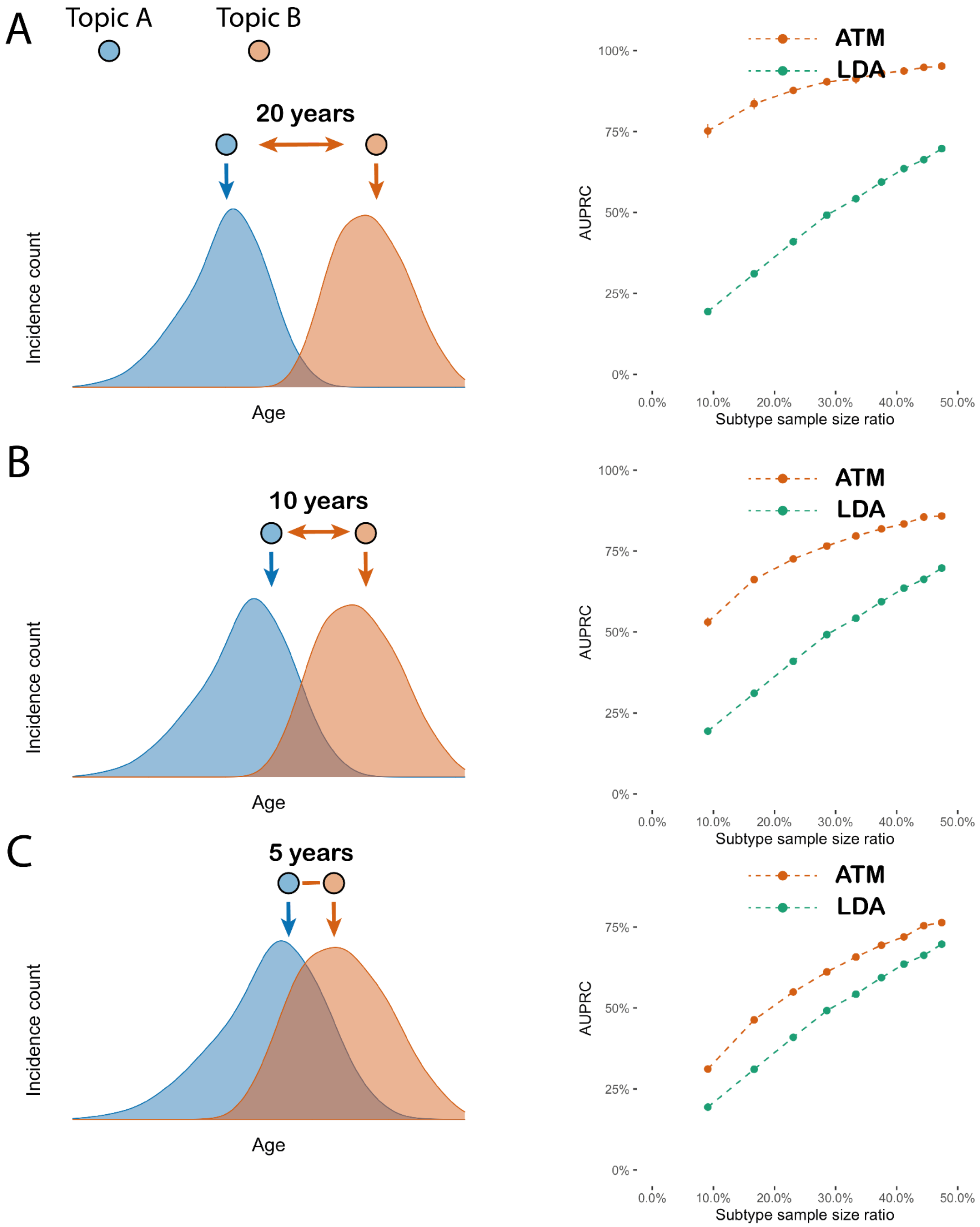

Supplementary Fig. 2. Same analysis as in Fig. 2 but simulating the smaller subtype to have older age at diagnosis. The area under precision and recall curve (AUPRC) to correctly assign incident disease to correct comorbidity profiles using Latent Dirichlet Allocation (LDA) and ATM. X-axis refers to the proportion of cases that belong to the older subtype (the orange subtype); precision and recall are computed for classifying the incidences in the older subgroup. Each dot represents the mean of 100 simulations of 10,000 people, the

bar shows the 95% confidence intervals. In the right column red refers to the ATM and green refers to the LDA model. Note AUPRC is only meaningful when precision and recall pertains to classifying the smaller subtype, therefore we simulate with the smaller subtype taking up to 50% of cases. (a) Scenario where two subtypes are simulated with 20 years of difference in age at diagnosis. (b) Scenario where two subtypes are simulated with 10 years of difference in age at diagnosis. (c) Scenario where two subtypes are simulated with 5 years of age difference.

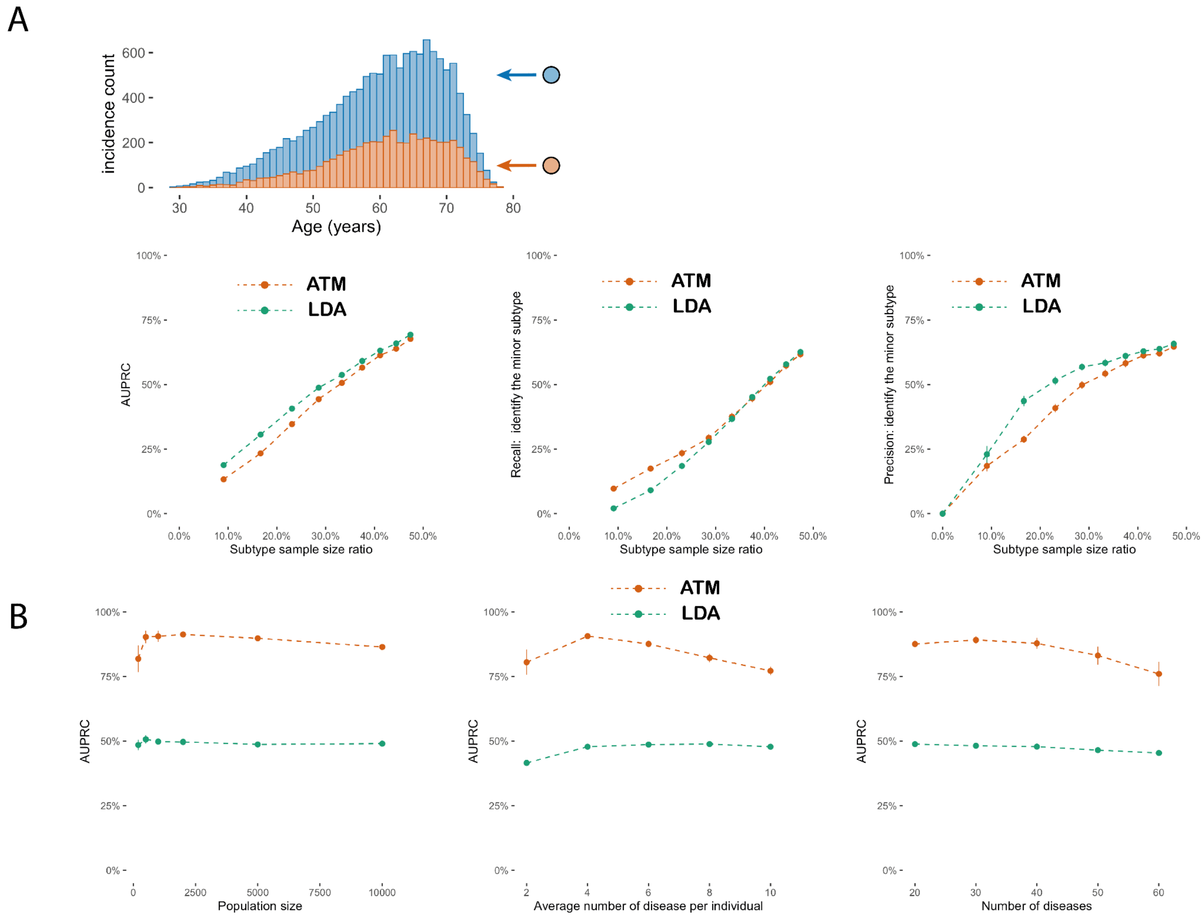

Supplementary Fig. 3. Additional simulation studies established the power of the method to identify comorbidity. (a) Same analysis as Fig. 2 but simulated subtypes with same age at diagnosis distribution. LDA outperforms ATM slightly as we have additional regularisation when modelling topic loading as functions of age, while for LDA age is not modelled. (b) AUPRC computed as in Fig. 2A with varying population size, average number of diseases per individual, and number of distinct diseases. Each dot shows the mean of 20 simulations and the bar shows 95% confidence interval.

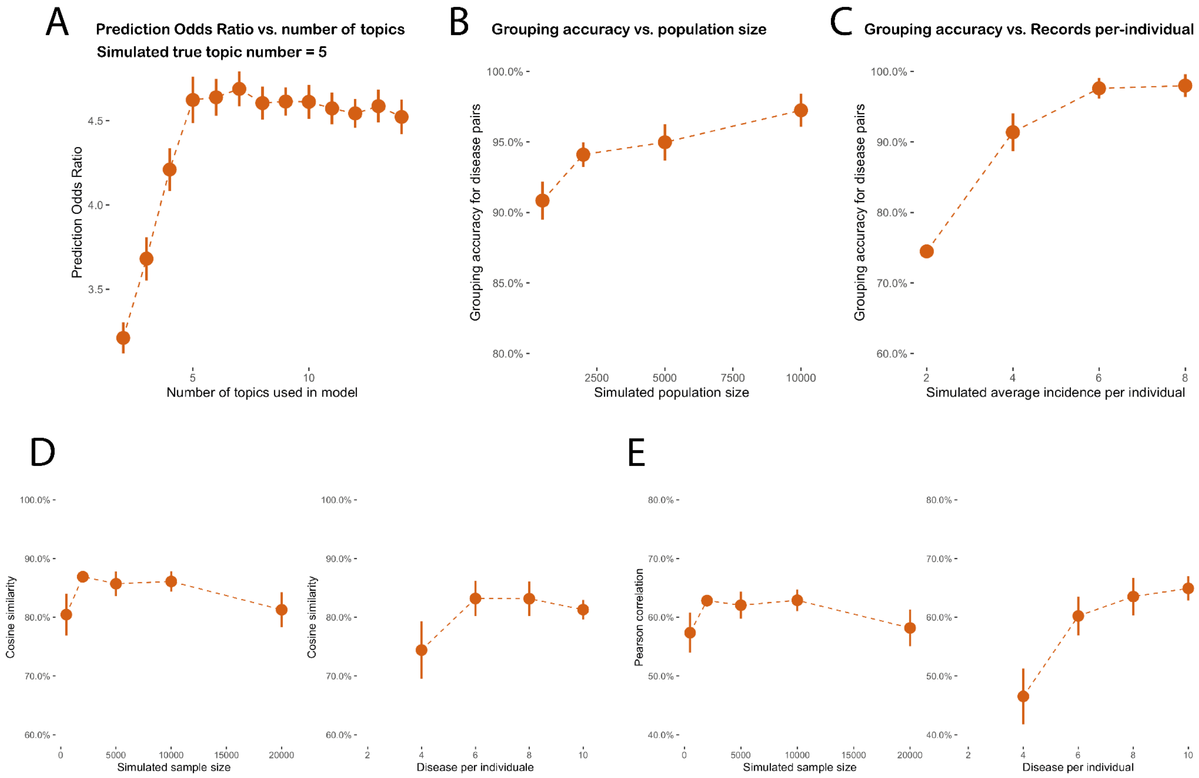

Supplementary Fig. 4. Simulations confirming that ATM could accurately recover topic loadings and topic weights. (a) We simulated data using 5 topics while fitting models of varying topic numbers. To compute prediction odds ratios (see Methods), we used 80% of data as training data to fit ATM and computed prediction odds ratio in the held out data, where we use the topic loading computed from the training data and prior diseases to infer the topic weights to predict the target diseases. The simulation was performed for 20 replications for each topic number in the inference. (b-c) We assign each disease to a single topic based on topic loading and compute the grouping accuracy as the proportion of disease pairs that are correctly grouped to the same topic. The grouping accuracy remains high for varying simulated population size and average disease per individual. (d) Recovery of topic loadings. We evaluate the accuracy of topic loading inference by computing the cosine similarity between inferred topic loading with the underlying truth. We match the inferred topics with the true topics using correlation of topic weights, using a greedy procedure (matching the first inferred topic from all true topics and then matching the next topic from the remaining not-matched true topics) to ensure the matching is bijectively. (e) Recovery of topic weights. We evaluate the accuracy of topic weight inference by computing the correlation of inferred topic weights and with the underlying truth. The ordering of topics uses the same strategy as in panel d. Points show the mean values across simulations and error bars are the 95% confidence intervals.

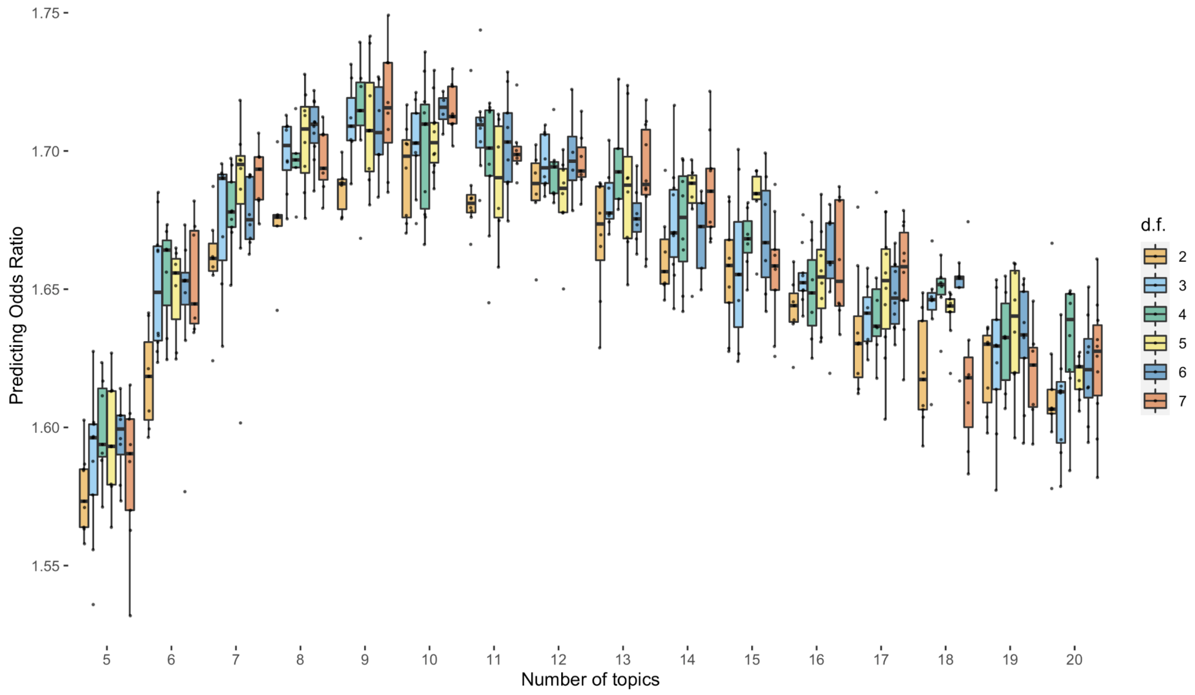

Supplementary Fig. 5. Prediction odds ratio across different model configurations. Each dot represents one inference on a random training and testing split of the UK Biobank individuals. The models are run with different topic numbers and parametric configurations of topic loadings. Degrees of freedom (d.f.) from 2 to 7 represent linear, quadratic polynomial, cubic polynomial, spline with one knot, spline with two knots, and spline with three knots.The prediction odds ratios are computed on the testing data using topic loadings inferred from the training data and topic weights inferred using previous diseases of testing individuals. The odds ratios are between the odds that target diseases are within model-predicted top percentile disease set versus the odds that target diseases are within the prevalence-ordered top percentile disease set. Box plots show the distributions of the dots; centre, box bonunds, and whisker ends denote median, quartiles, and minima/maxima.

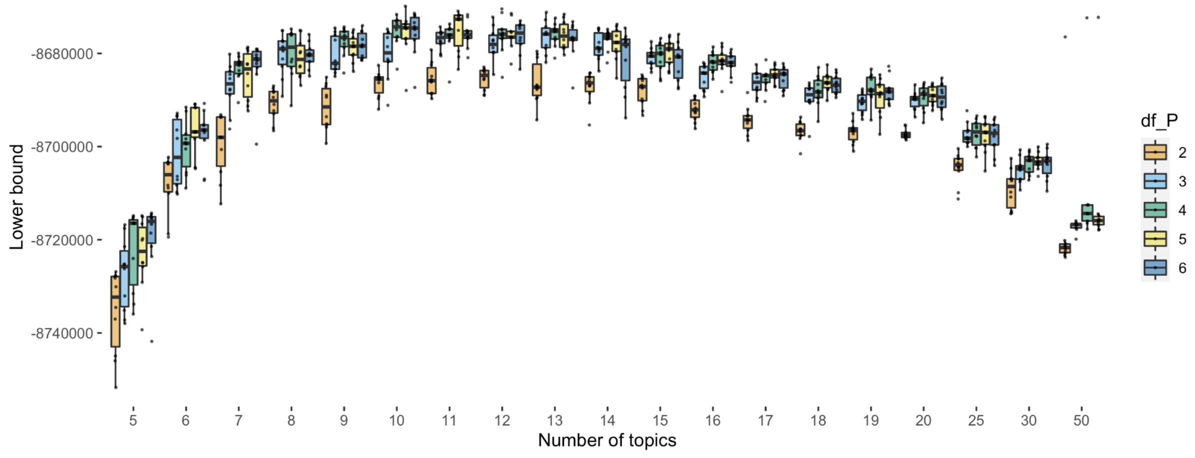

Supplementary Fig. 6. Evidence lower bound (ELBO) of different model configurations on the entire dataset. Each dot represents one inference on a random training and testing split of the UK Biobank individuals. The models are run with different topic numbers and parametric configurations of topic loadings. Degrees of freedom (d.f.) from 2 to 7 represent linear, quadratic polynomial, cubic polynomial, spline with one knot, spline with two knots, and spline with three knots. Box plots show the distributions of the dots; centre, box bonunds, and whisker ends denote median, quartiles, and minima/maxima.

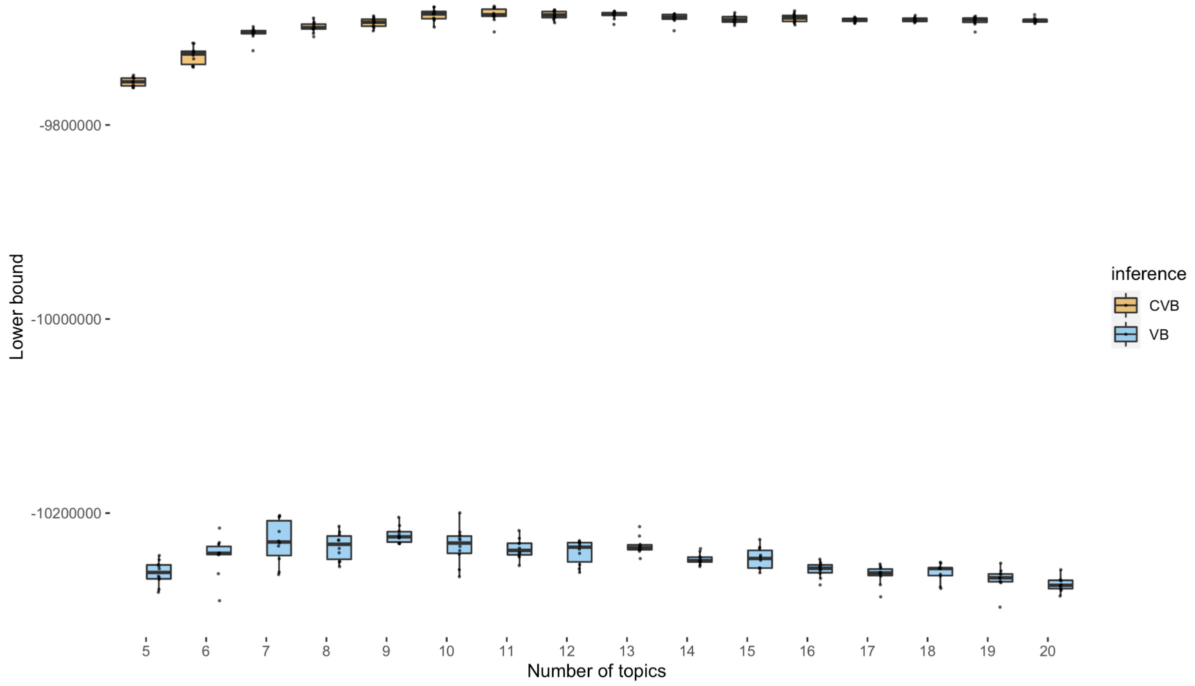

Supplementary Fig. 7. Comparison of ELBO for collapsed variational inference and mean-field variational inference. ELBO is computed by fitting the ATM using two inference methods on the entire UK Biobank dataset, where the topic loadings are configured as cubic polynomials. Models of different numbers of topics are fitted with 10 random initialisations for both CVB and the VB (mean-field variational inference, which is a more

commonly used inference method for Bayesian models). The ELBO of an inference method is a lower bound that approximates the evidence function, which depends on the number of topics and parametric form of topic loading, but not the inference methods; higher ELBO means better inference accuracy. Box plots show the distributions of the dots; centre, box bonunds, and whisker ends denote median, quartiles, and minima/maxima.

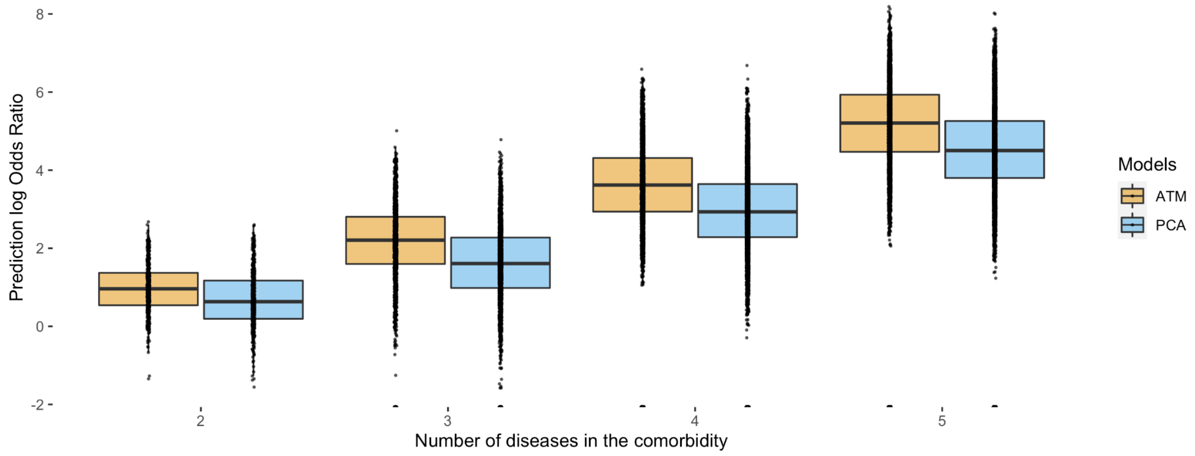

Supplementary Fig. 8. Prediction log odds ratio of comorbidities. Diseases combinations (comorbidities) are extracted from topics loadings that are trained on a training set, using max value of average topic assignments (Methods). Roughly, the odds ratio of each disease combination is computed by selecting all disease sets containing combinations of 2, 3, 4, and 5 diseases assigned to the same topic by max topic loading, and dividing incidences where the disease sets appeared in one patient by the expected number in an independent testing set. We show the comparison of ATM and PCA for all combinations of 2, 3, 4, and 5 diseases; here we use PCA as we wish to show the superiority of topic modelling in identifying clusters of disease compared to other low-rank methods that are not based on multinomial distribution. Box plots show the distributions of the dots; centre, box bonunds, and whisker ends denote median, quartiles, and minima/maxima.

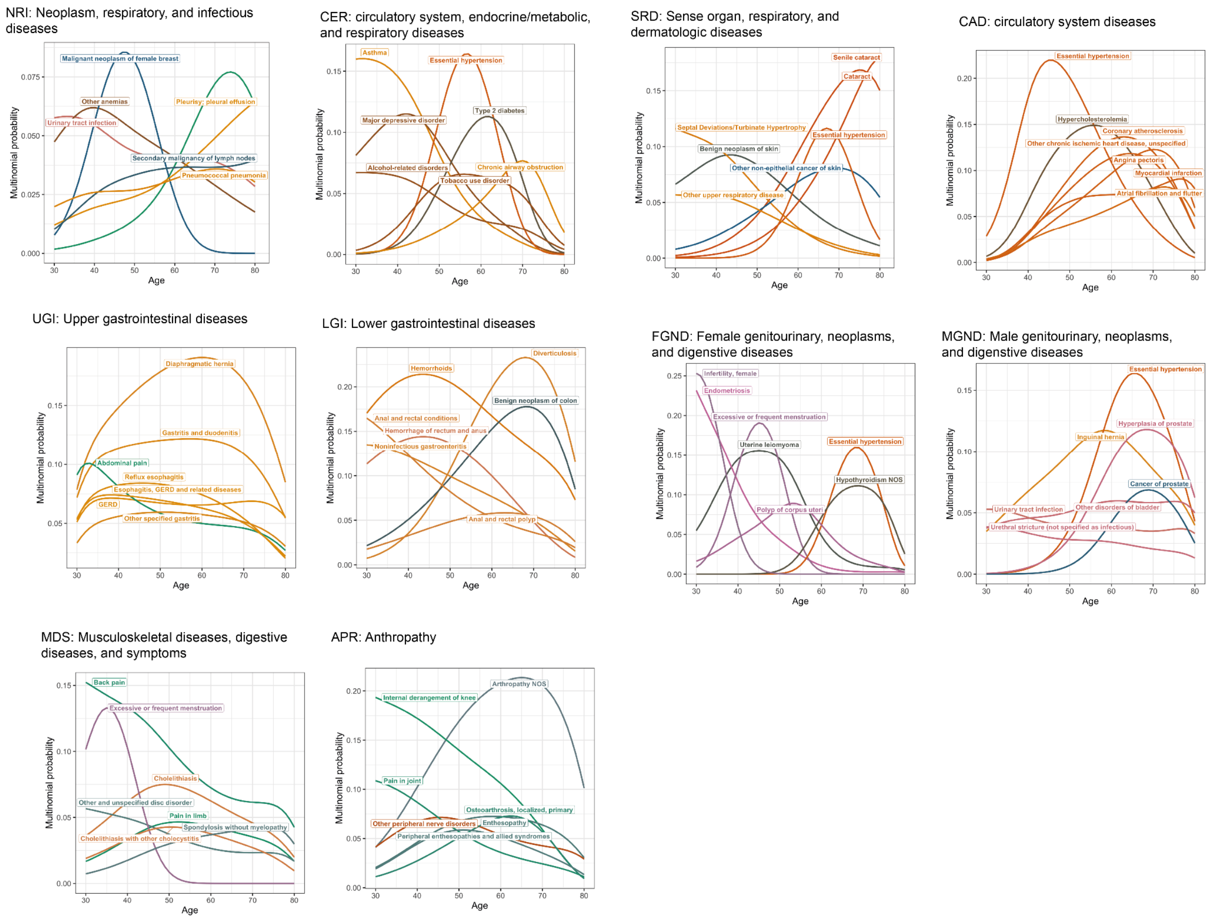

Supplementary Fig. 9. Top seven diseases in each comorbidity topic. Seven diseases that have highest loading within the topic are shown for each comorbidity topic. Colour of the curves reflect the ordering of Phecodes. We chose seven for best visual presentation. Numerical results are reported in Supplementary Table 5.

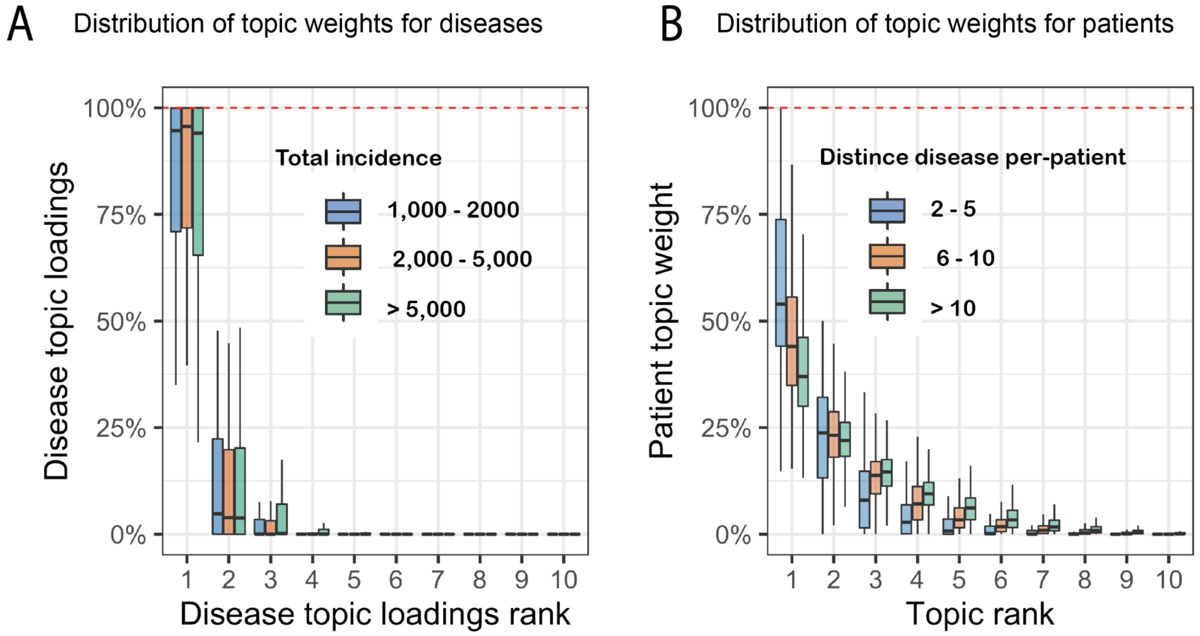

Supplementary Fig. 10. Additional topic sparsity analysis. (a) Sparsity of disease topic loadings. Box plot shows the distribution of topic loading for disease of different incidence numbers. (b) Sparsity of patient topic weights. Box plot shows the topic weight distribution in decreasing order for individuals with different numbers of diagnosis. Centre, box bonunds, and whisker ends denote median, quartiles, and minima/maxima.

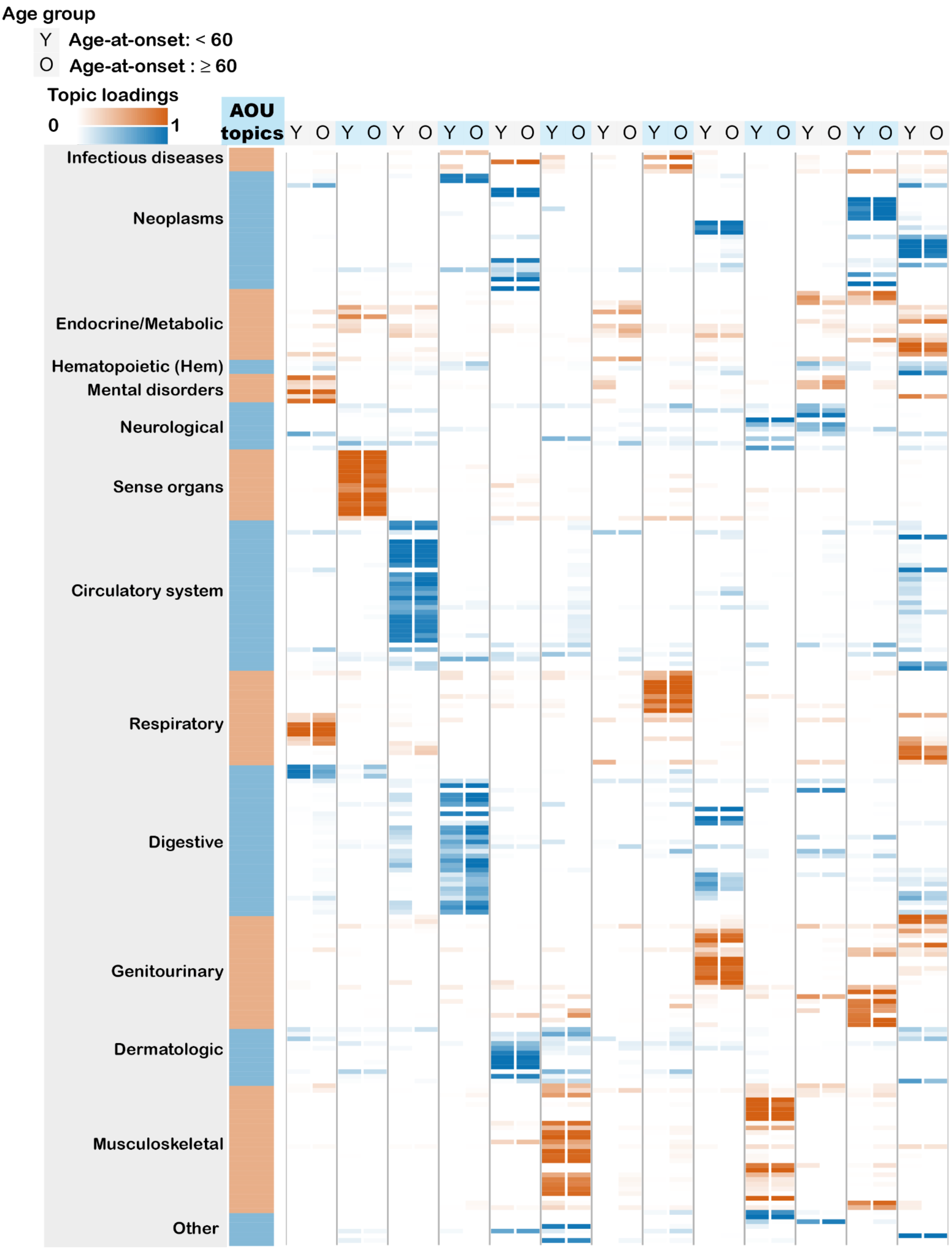

Supplementary Fig. 11. Age-dependent topic loadings of 13 inferred disease topics across 233 diseases in the All of Us. We report topic loadings averaged across younger ages (age at diagnosis < 60) and older ages (age at diagnosis > 60). Row labels denote disease categories ordered by Phecode systems, with alternating blue and red color for visualisation purposes; "Other" is a merge of five Phecode systems: "congenital anomalies", "symptoms", "injuries & poisoning", "other tests", and "death" (which is treated as an additional disease, see Methods). Topics are ordered by the corresponding Phecode system. This figure is an All of Us equivalent of Fig. 3.

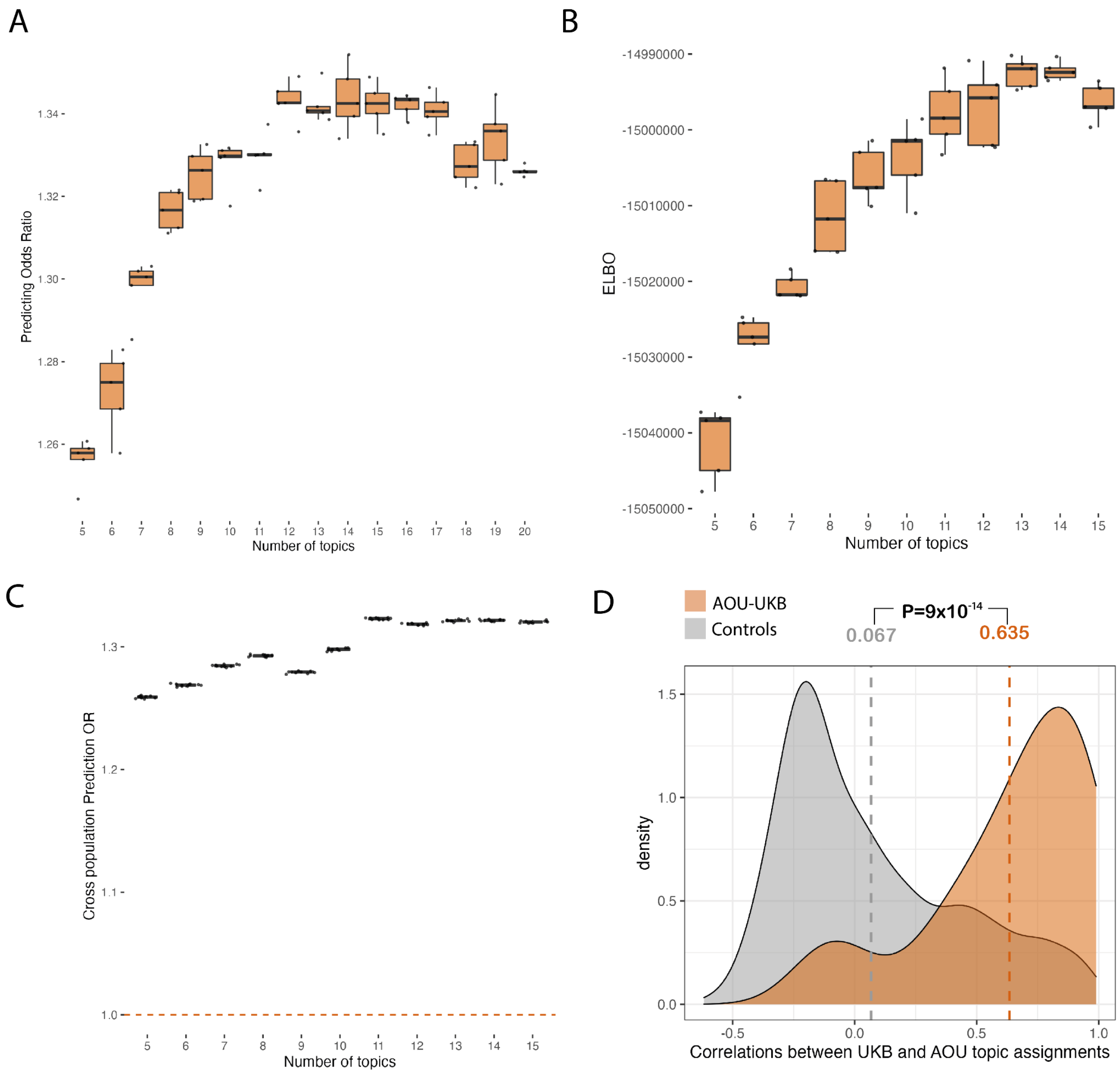

Supplementary Fig. 12. ATM infers disease topics from All of Us cohort which align with topics from UK Biobank. (A) Prediction odds ratio using ATM model with different topic numbers in All of Us. Each dot represents one of the five-fold cross validation within the All of Us individuals. (B) Evidence lower bound (ELBO) of different ATM model configurations on the entire All of Us dataset. Each dot represents one inference with random initialization. The models are run with different topic numbers and same configurations of topic loadings (spline model with one knot). (C) Prediction odds ratio on UK Biobank

individuals using All of Us topic loadings. We divide the UKBiobank population into 10 jackknife blocks and each dot represents the prediction odds ratio on one leave-one-out jackknife sample. Topic weights are inferred using prior diseases of UKBB individuals, using loadings trained from All of Us. The odds ratios are between the odds that target diseases are within model-predicted top two-percentile disease set versus the odds that target diseases are within the prevalence-ordered top two-percentile disease set. Prediction odds ratio is 1.32 (s.e. = 0.0027) when using the optimal 13 All of Us topic to predict UK Biobank diagnoses. We chose the top-two percentile to match the UK Biobank analysis as All of Us has 233 of the 348 diseases analysed in the UK Biobank. (D) Correlations between UKB and AOU topic assignments for 41 diseases with subtypes between AOU and UKB (red shade) are significantly higher than expected (grey shade). The correlation between 41 AOU-UKB disease pairs are reported in Table 2 and Extended Data Fig. 5. Grey shade is the distribution of non-diagonal correlations in Extended Data Fig. 5. Grey and red vertical dashed line reports the mean of the grey and red shades; P-value is for a two-sided t-test of the difference of the mean. Box plots show the distributions of the dots; centre, box bonunds, and whisker ends denote median, quartiles, and minima/maxima.

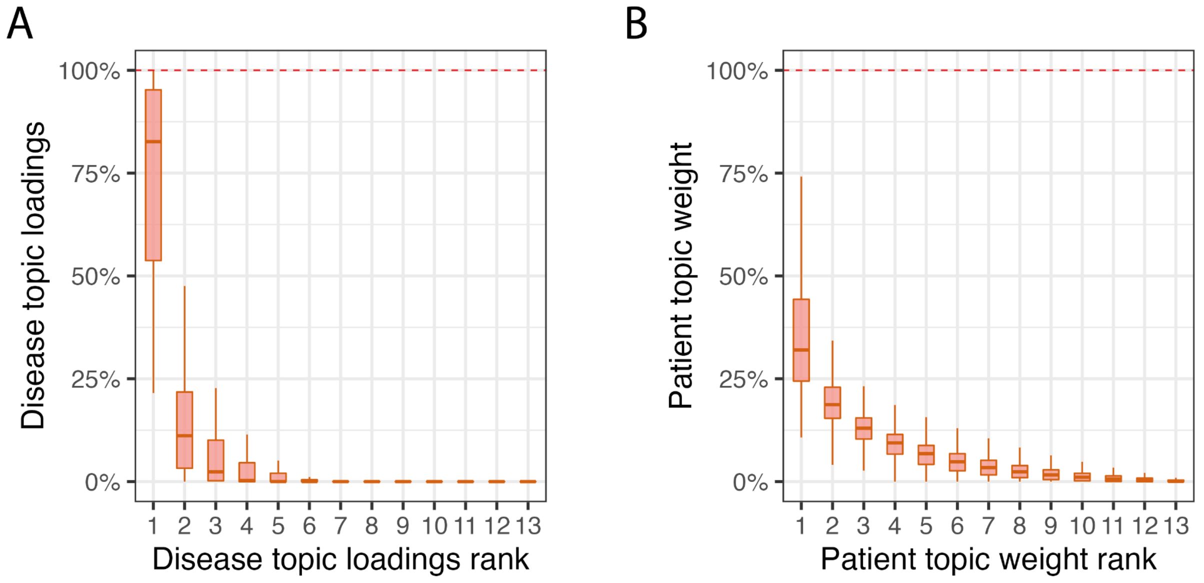

Supplementary Fig. 13. Distribution of topic loading across diseases and topic weights across patients for All of Us. (A) Box plot of disease topic loading as a function of rank; disease topic loadings are computed as a weighted average across all values of age at diagnosis. (B) Box plot of patient topic weight as a function of rank. Centre, box bonunds, and whisker ends denote median, quartiles, and minima/maxima. This figure is an All of Us equivalent of Fig. 4B-C.

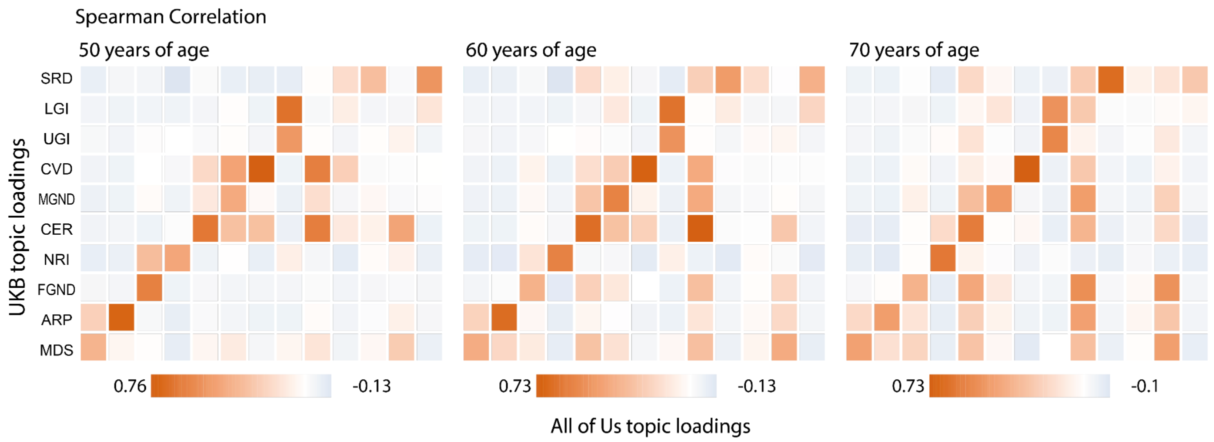

Supplementary Fig. 14. correlation between topic loadings from UK Bibank (y-axis) and All of Us (x-axis) for three age slices. The figures are the age-specific versions for Fig. 5C.

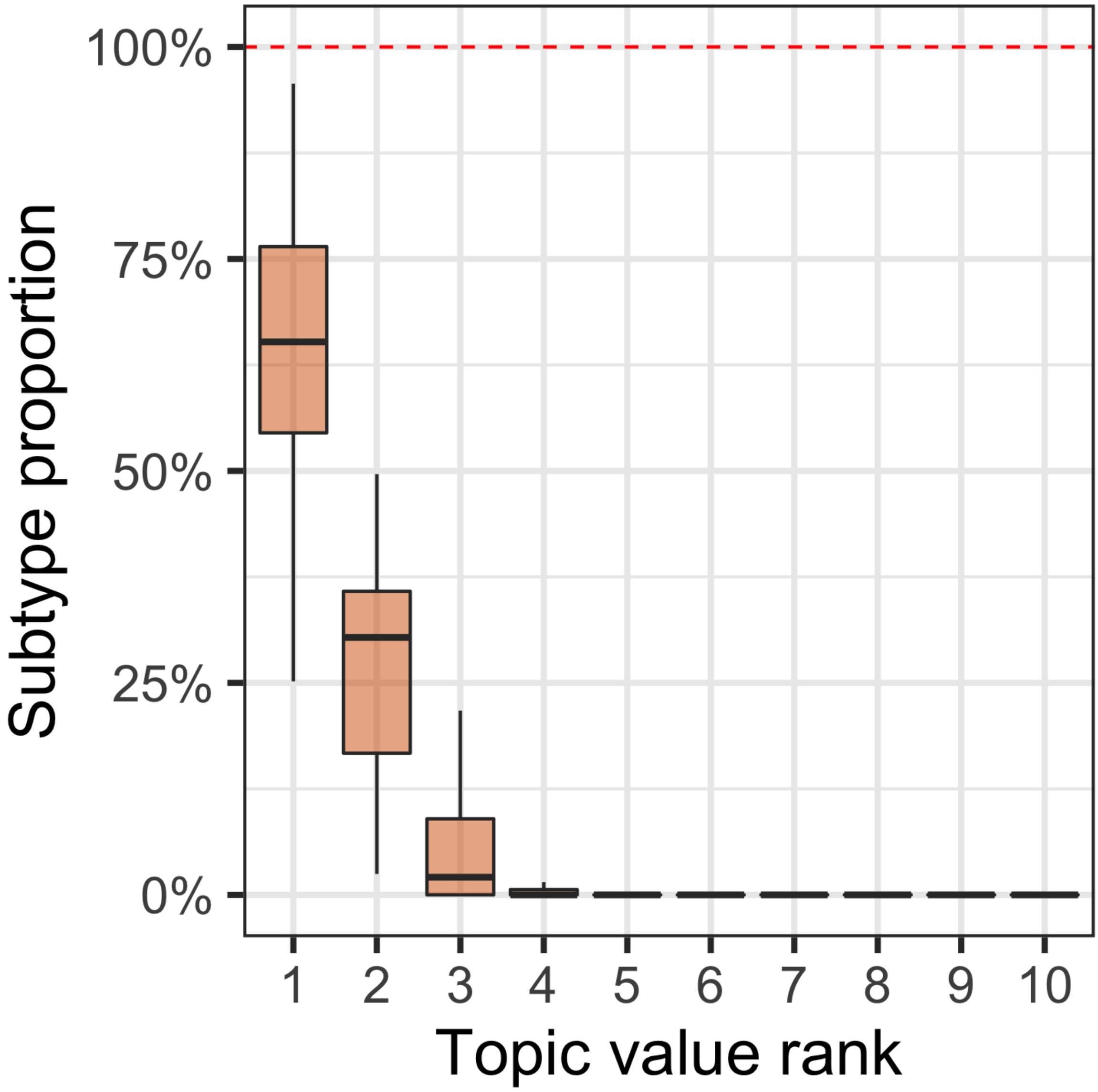

Supplementary Fig. 15. Topic distribution for the 52 diseases that have at least 500 cases assigned to distinct topics. For each disease, we computed the average topic assignments as the proportion of diagnoses assigned to each subtype. The box plot shows the distribution of the subtype proportion from the largest (leftmost boxes) to the smallest; centre, box bonunds, and whisker ends denote median, quartiles, and minima/maxima. For nearly all diseases, the cases are concentrated into three subtypes, with very few cases assigned to other topics.

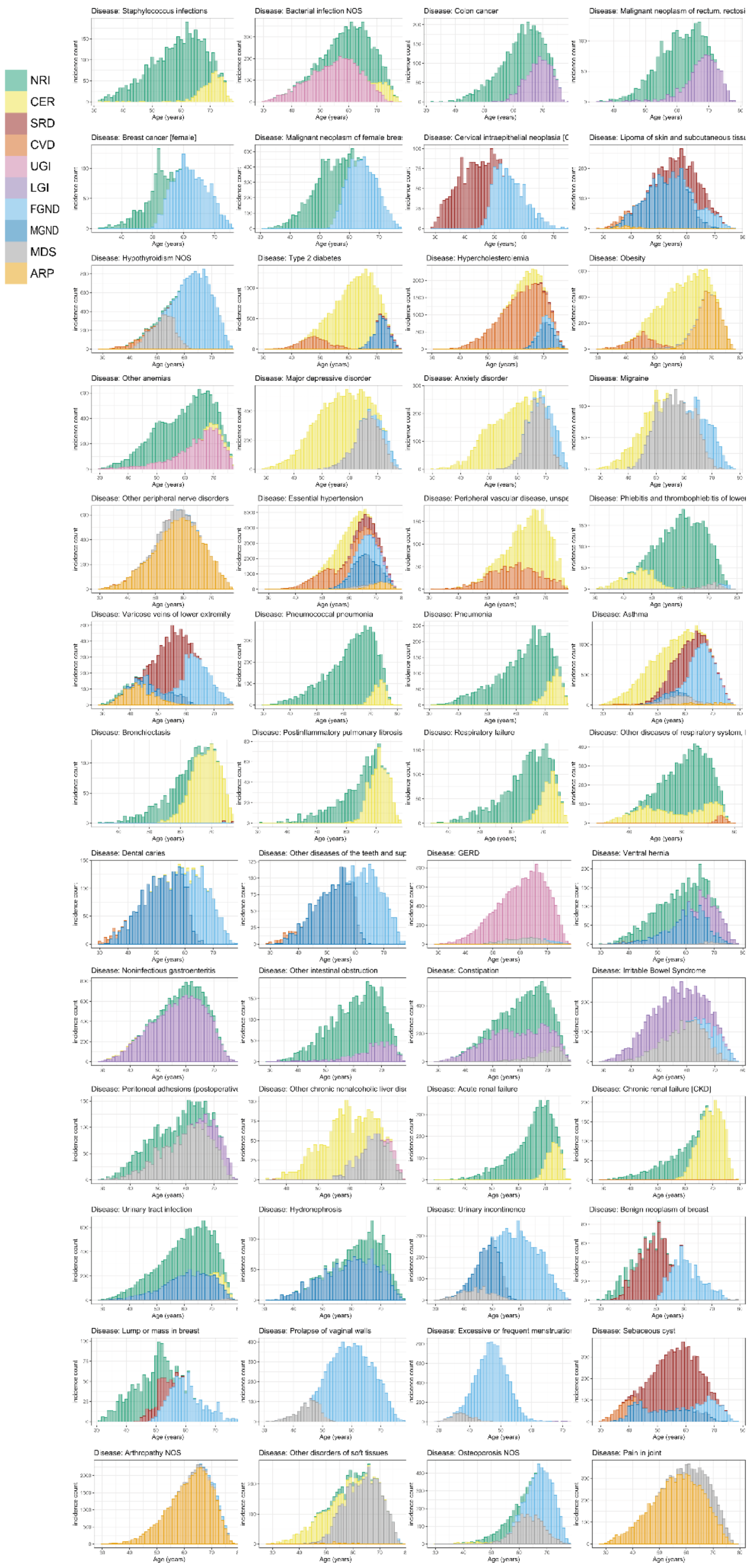

Supplementary Fig. 16. Comorbidity subtype distribution over age for 52 diseases. Diseases shown are ordered by 13 phecode systems: infectious diseases, neoplasms, endocrine/metabolic, hematopoietic, mental disorders, neurological, circulatory system,

respiratory, digestive, neoplasms, genitourinary, dermatologic, and musculoskeletal. Numerical results are reported in Supplementary Table 11-12.

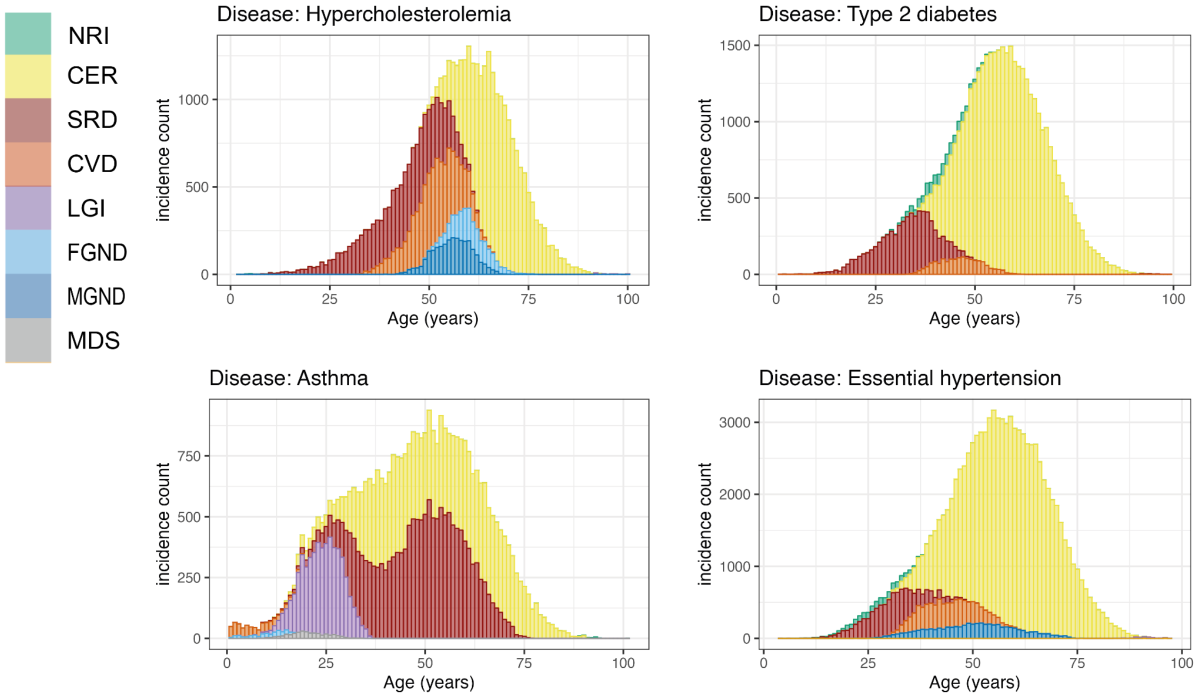

Supplementary Fig. 17. Stacked bar plots of age-dependent subtypes in All of Us. Disease topics in All of Us are mapped to their most similar UK Biobank topics; colours are the same as Supplementary Fig. 16. The figures are for 4 representative diseases in Fig. 6A (type 2 diabetes, asthma, hypercholesterolemia, and essential hypertension); for each disease, we include all subtypes with at least one diagnosis.

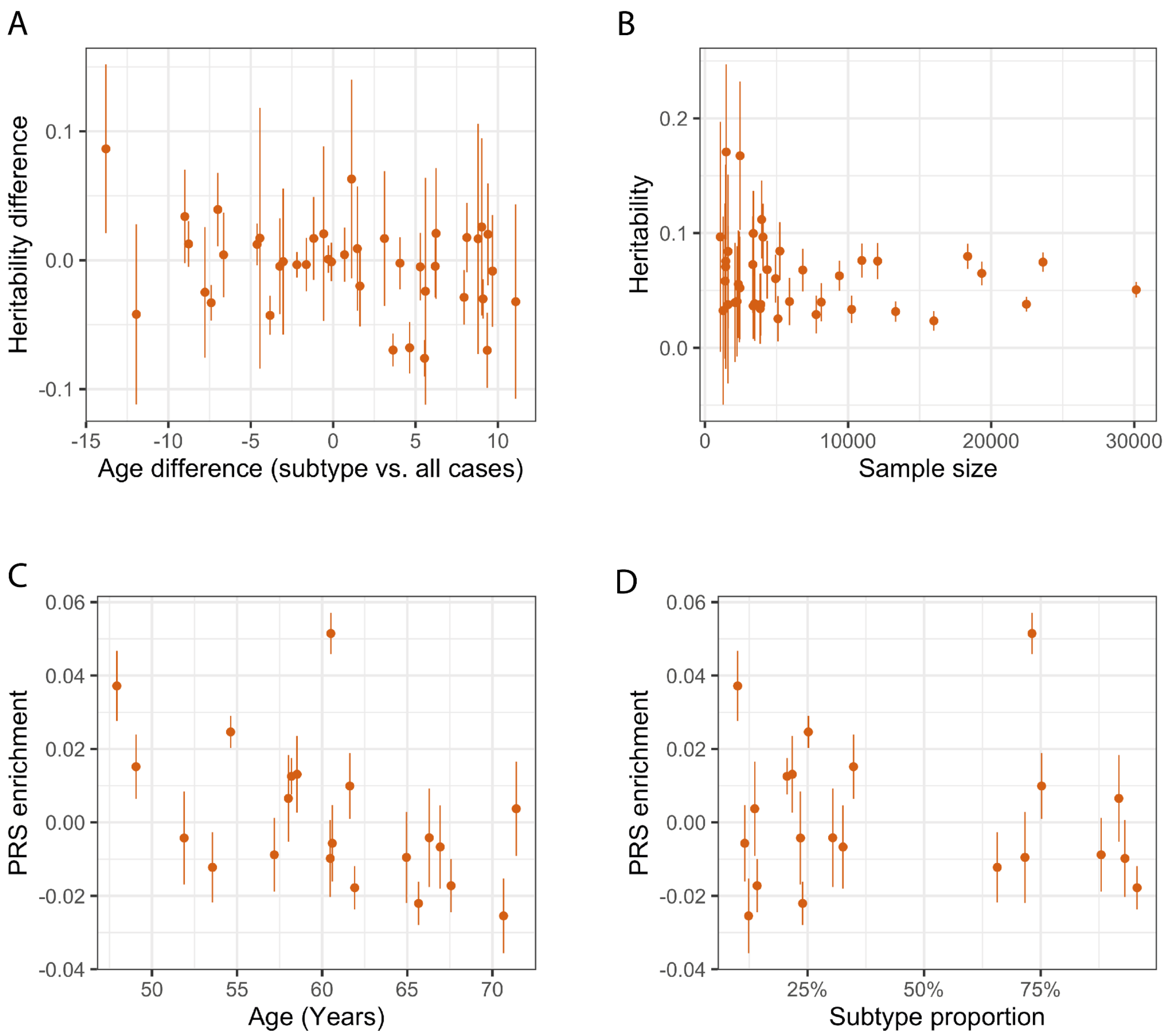

Supplementary Fig. 18. Heritability and PRS are not associated with age and subtype size. (a) We plot heritability deviation from all cases versus age deviation from all cases of 41 subtypes (from 14 diseases that have heritability z-score above 5). The heritability is estimated by first performing mixed-effect association analysis using BOLT-LMM on imputed SNPs from the British Isle Ancestry then using LDSC. (b) Heritability for the subtypes plotted with the sample size of the subtype. (c-d) Excess PRS from Fig. 6B plotted against the age and the sample size (denoted by the ratio of samples between subtype and all cases) for the subtypes. The dots and the bars show the mean and 95% confidence interval across all subfigures.

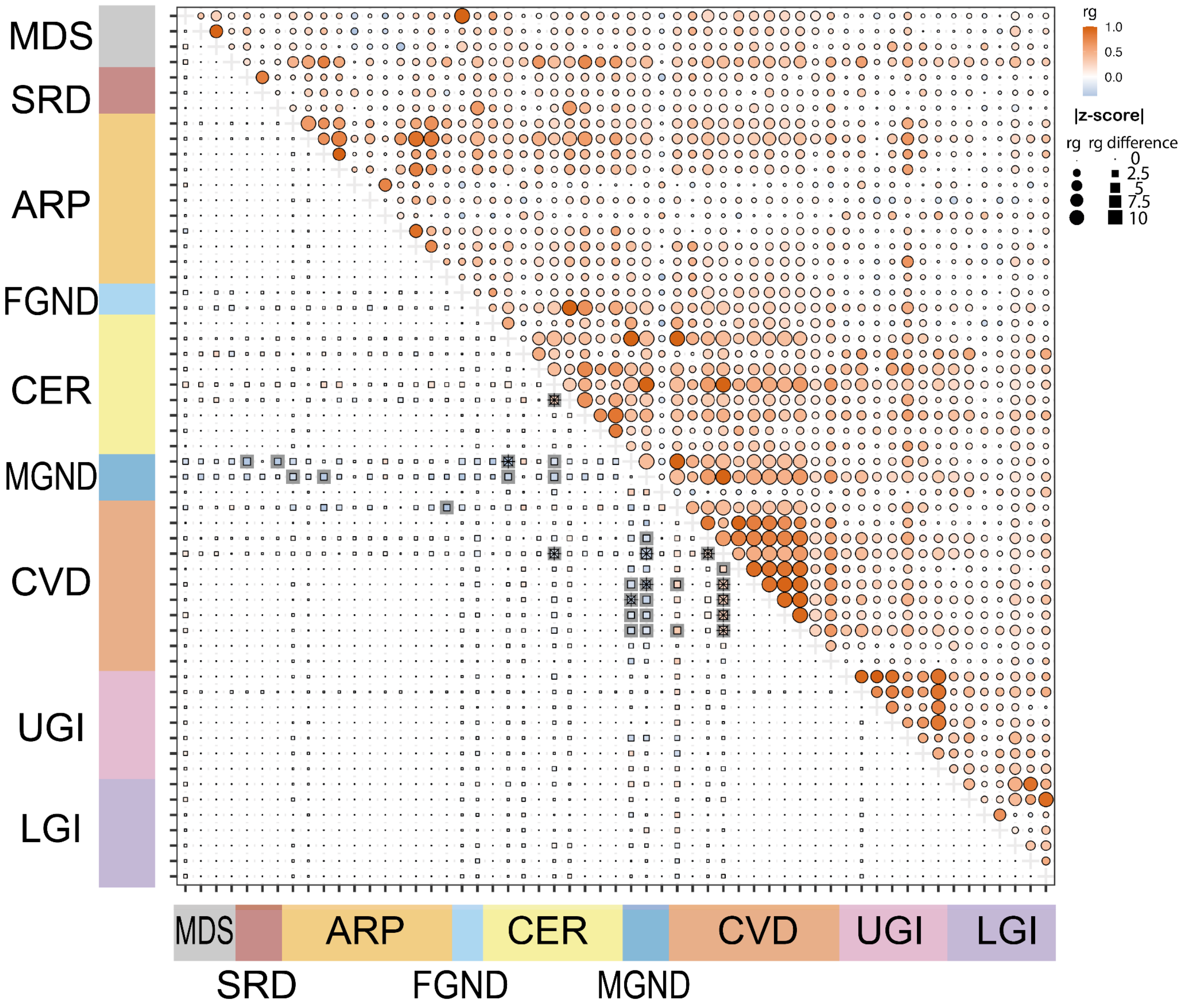

Supplementary Fig. 19. Excess genetic correlation (r_g) between disease subtypes across disease subtypes. Lower left panel shows the r_g of disease-subtype or subtype-subtype pairs subtracted by the r_g of corresponding disease-disease pairs. Each row or column represents a disease or subtype. For disease-disease pairs, the excess r_g is not defined in the lower left panel, since the difference is 0. The upper right panel shows r_g of corresponding disease-disease pairs, where values could be duplicated as the same disease could have multiple subtypes. The 89 diseases or subtypes are chosen here by heritability z-score > 4 and r_g z-score > 4 with at least one other disease or subtypes. We kept 57 rows and columns for better visualisation by removing 32 diseases that have subtypes included. A star means FDR < 0.1, while a shade means a nominal statistical significance at P = 0.05 (for a z-score test).

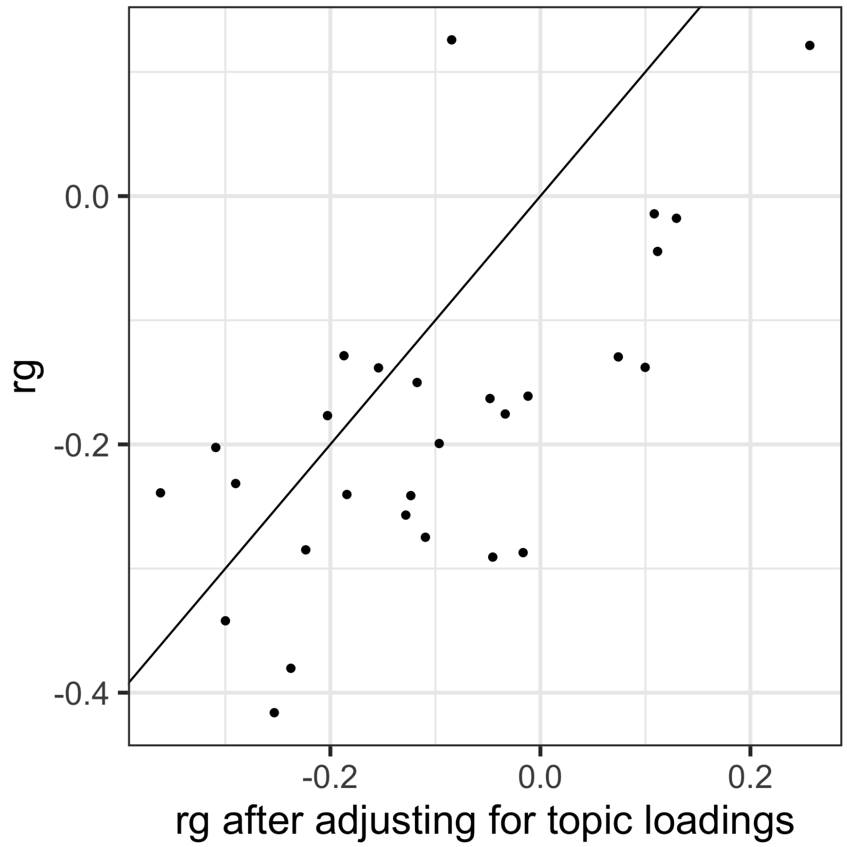

Supplementary Fig. 20. Comparison of excess genetic correlation (r_g) between subtypes using summary statistics from case-control matched by topic weights (x-axis) and not matched by topic weights. The analysis is performed on subtypes of four diseases: type 2 diabetes, hypercholesterolemia, hypertension, and asthma. The excess r_g (shown in panel (A) of Fig. 7) of two case-control matching strategies across 28 subtype pairs are compared and the effect lies along the diagonal line. The excess genetic correlation attenuated slightly when topic weights are matched, while it can not explain all the excess r_g.

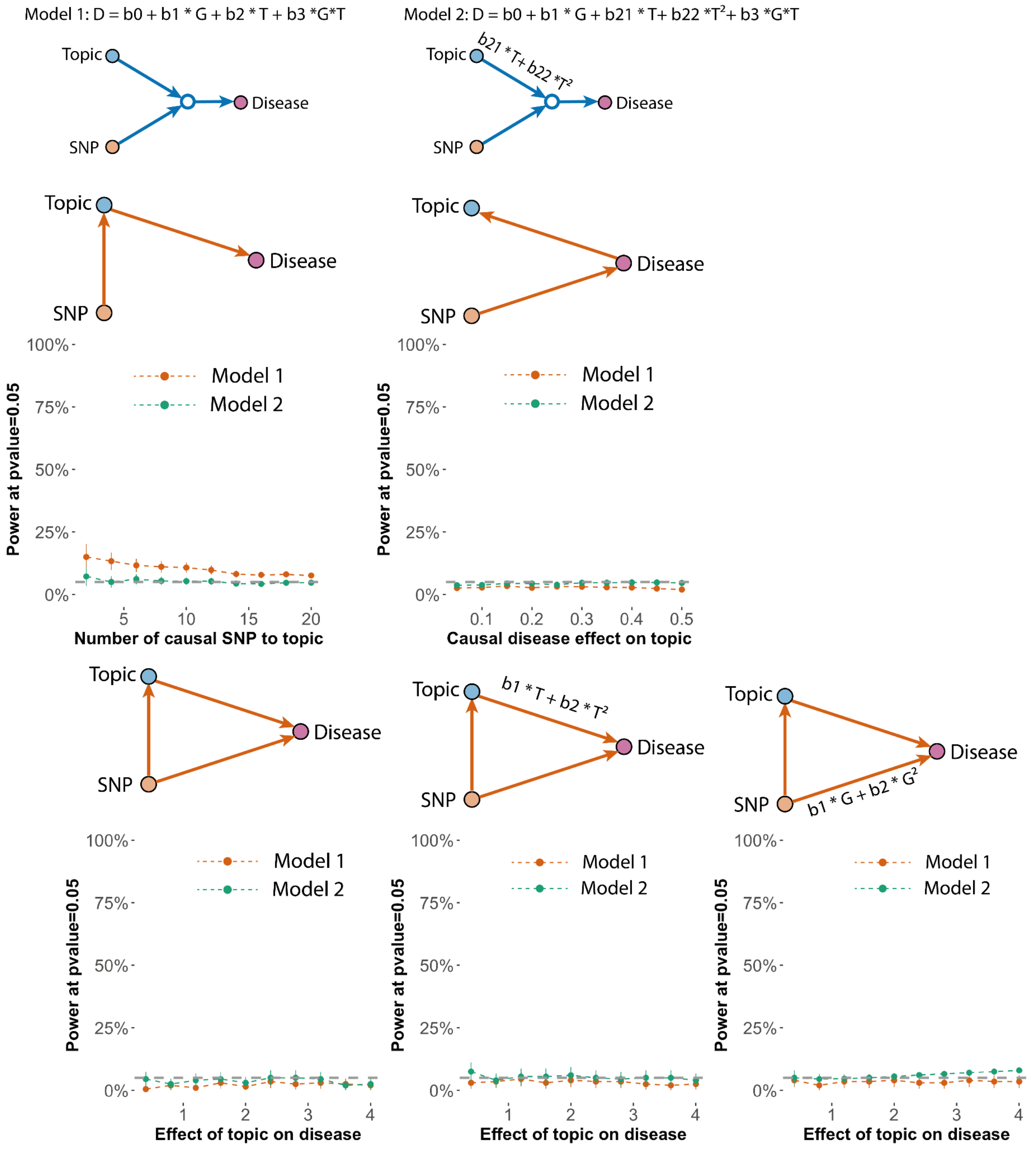

Supplementary Fig. 21. Simulation analysis verified the SNP x topic interaction tests are calibrated when no actual interaction exists. We show the false positive rate of two models under five simulated model structures where no actual interaction exists (Methods). The false positive rate is computed as the power to detect interaction effects using model 1 (red; linear model) and model 2 (green; with non-linear main effect term) under P-value=0.05. The five structures evaluated are (1) SNP causal to topic and topic causal to disease; (2) SNP causal to disease and disease causal to topic; (3) SNP is causal to both topic and disease; (4) and (5) SNP is causal to both topic and disease with nonlinear effects. Genotypes are simulated using the MAF from the 888 disease associated SNPs that were analysed in the SNPxTopic interaction tests. Points show the mean values across simulations and error bars are the 95% confidence intervals; all tests are for the interaction regression coefficients.

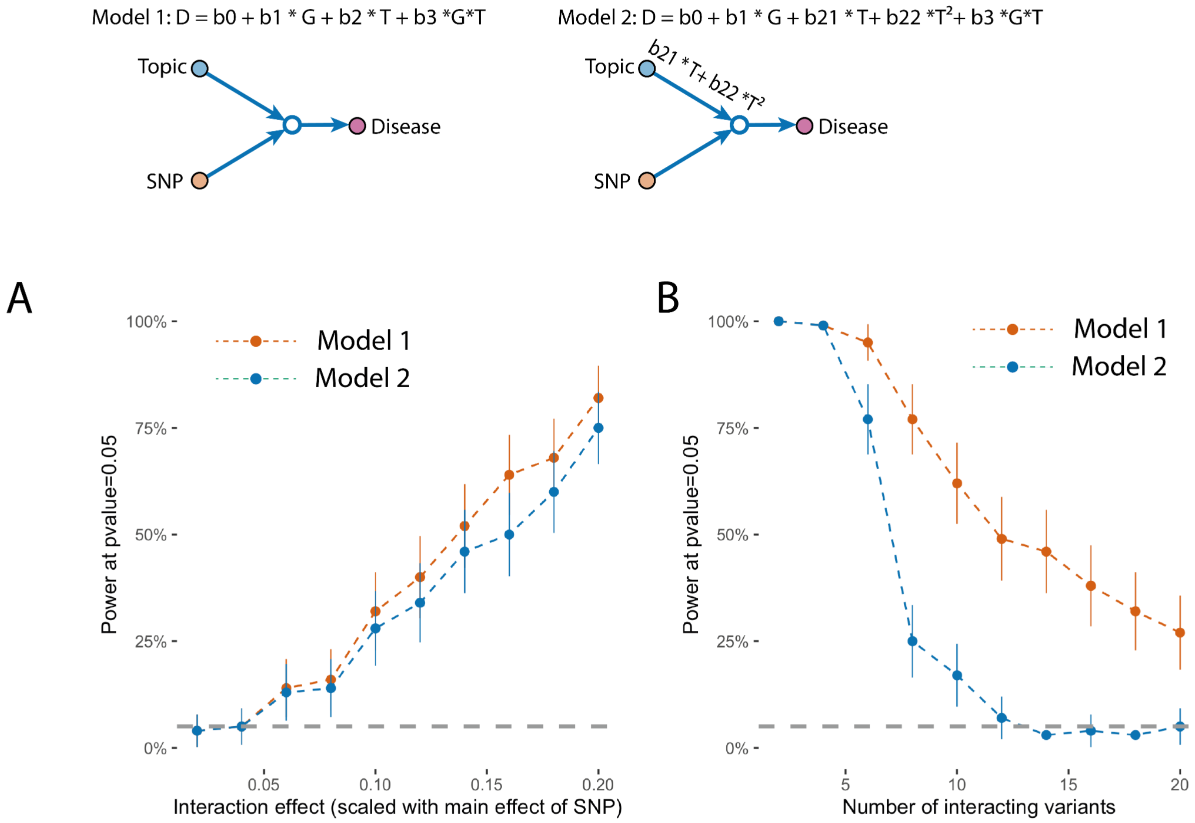

Supplementary Fig. 22: Power to detect true SNP x topic interaction effect under simulation. (a) We simulated data with 10,000 individuals, topic-to-disease main effect size equal to 2, interaction effect from 0.04 to 0.4, and SNP-to-disease main effect size proportional to the interaction effect (0.02 to 0.2); disease diagnoses are generated using gaussian liability with top 20 percentile as cases. We tested for the SNP x topic interaction using model 1 and model 2 and computed the power of discovering the true interaction. (b) We simulated data with 10,000 individuals and an interaction effect equal to 0.4. Instead of simulating a single SNP effect, we simulated 2 to 20 variants that all interact with topic weight. We then test the SNP x Topic interaction in model 1 and model 2 with one variant at a time, which is the same strategy as most GWAS interaction tests. We note the power of model 2 is lower than model 1, while we still choose model 2 as it is better calibrated (Supplementary Fig. 21). Points show the mean values across simulation and error bars are the 95% confidence intervals.

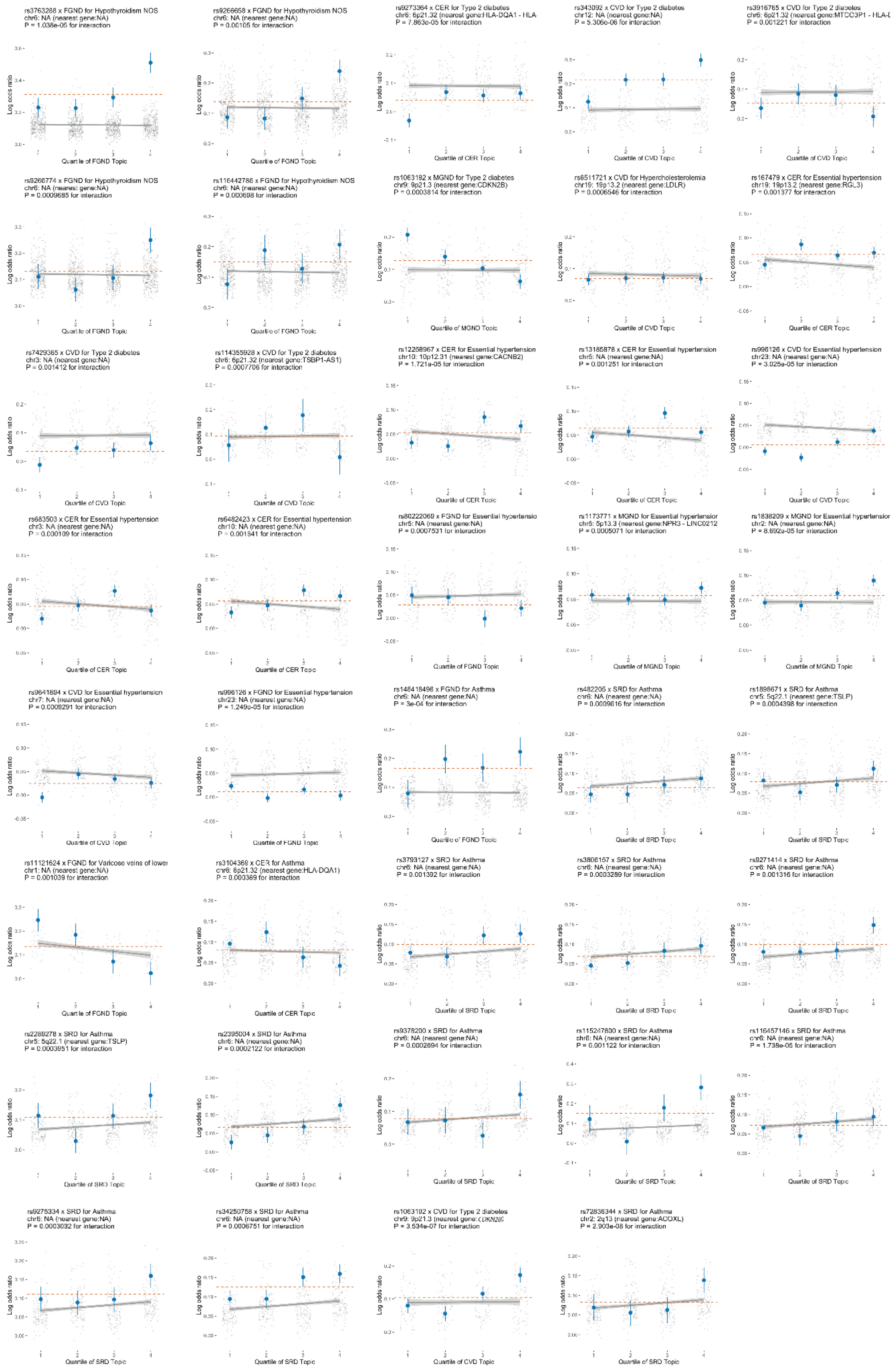

Supplementary Fig. 23. Additional 39 SNPs (mapped genes in the parentheses) that have different effect sizes in different quantiles of topic weights. For each example, we report main SNP effects (log odds ratios) specific to each quartile of topic weights across

individuals, for both the focal SNP (blue dots) and background SNPs for that disease and topic (genome-wide significant main effect ($P < 5\times10^{-8}$) but non-significant SNP x topic interaction effect ($P > 0.05$); grey dots). Dashed red lines denote aggregate main SNP effects for each focal SNP. Error bars denote 95% confidence intervals. Grey lines denote linear regression of grey dots, with grey shading denoting corresponding 95% confidence intervals. P-values for interaction are for testing the interaction regression coefficients; P-values for top/bottom differences are for two-sided t-test. Numerical results are reported in Supplementary Table 19.

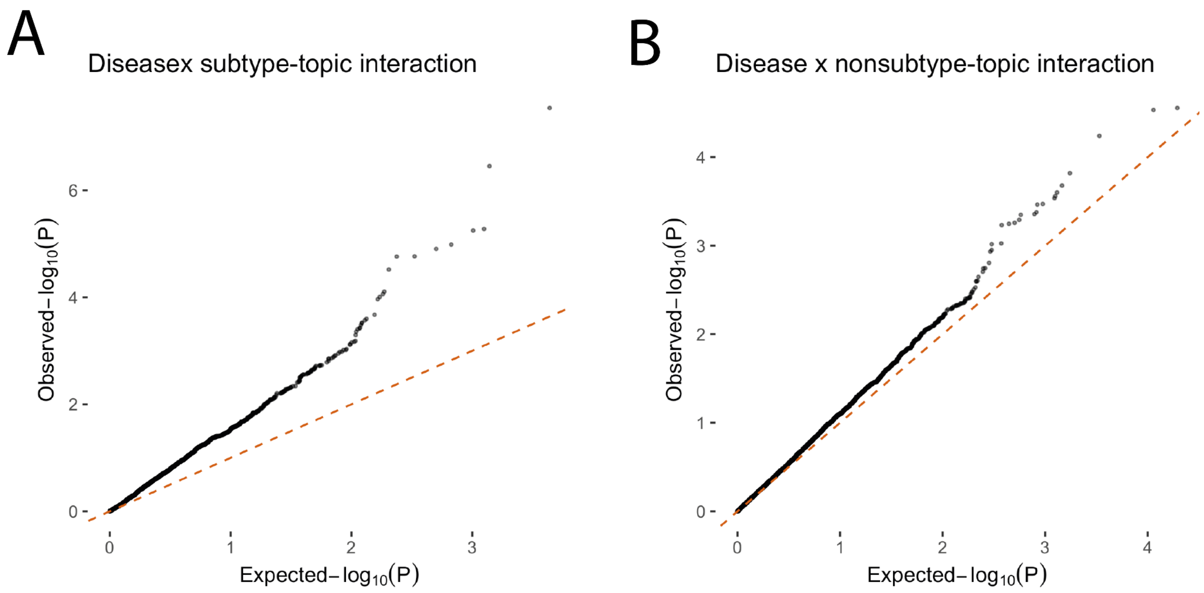

Supplementary Fig. 24: QQ plot of SNP x topic interaction for all GWAS SNPs ($P < 5\times10^{-8}$). (a) We show the interaction between SNP-topic where the topics define disease subtypes. We focus on the subset of subtypes whose disease have h^2 z-score larger than 4 to ensure there is enough GWAS signal for testing. The P-values are for testing the interaction effects with nonlinear topic-to-disease main effects (Model 2 in Supplementary Fig. 21). The median for observed p-value is 0.35. (b) As a control to show the calibration of the tests, we plot the QQ-plot over the same set of GWAS SNPs, but over the topic that are not identified as subtypes of the disease by ATM. The median for observed p-value in the Null test is 0.47. The observed small inflation of test statistics (0.47 < 0.5) is caused by the correlation between topics (i.e. a SNP that interacts with a subtype-topic is expected to have weak interaction with other non-subtype topics as the topic weights sum to one).

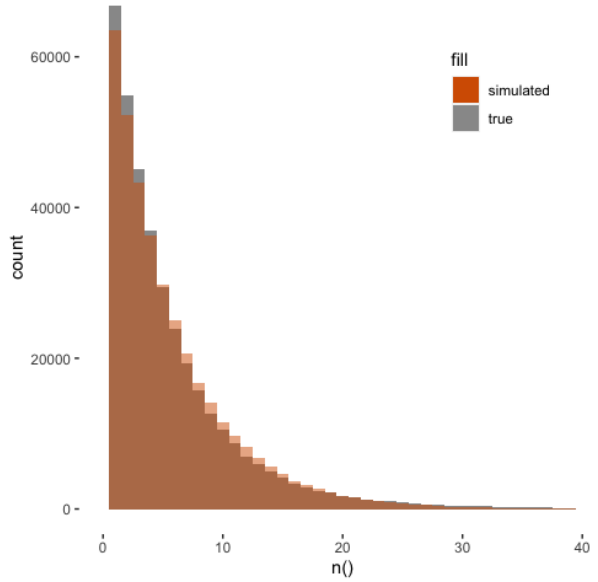

Supplementary Fig. 25. Comparison of simulated diagnosis per individual versus true UK Biobank data. Histogram of number of distinct diseases per patient from the UK Biobank HES dataset and from the simulated exponential distribution with mean = 6.1.

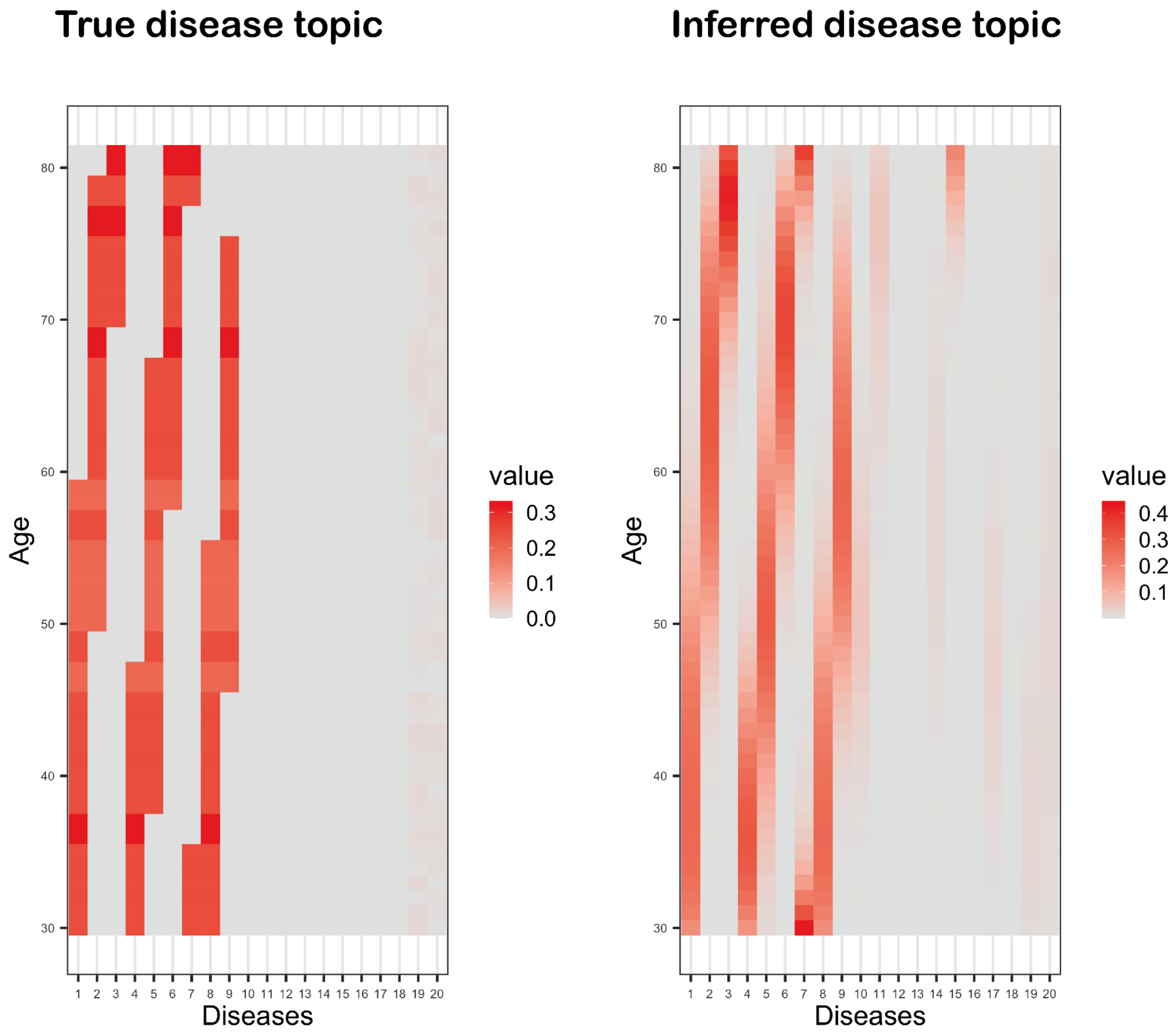

Supplementary Fig. 26. Examples of simulated vs. inferred topic loadings from ATM. The left panel shows the topic loadings used to simulate 10,000 individuals; the right panel shows the inferred topic loadings using topic loadings parametrized as cubic polynomials.
