# Supplementary material for: Age-dependent topic modeling of comorbidities in UK Biobank identifies disease subtypes with differential genetic risk
Source: Nat Genet. 2023 Oct 9;55(11):1854–65. doi: 10.1038/s41588-023-01522-8 (PMC10632146; doi:10.1038/s41588-023-01522-8)
Supplement: Supplementary file 2 — Reporting Summary [file 41588_2023_1522_MOESM2_ESM.pdf]

nature portfolio

Corresponding author(s): Gil McVean, Alkes Price, Xilin Jiang

Last updated by author(s): Nov 25, 2022

Reporting Summary

Nature Portfolio wishes to improve the reproducibility of the work that we publish. This form provides structure for consistency and transparency in reporting. For further information on Nature Portfolio policies, see our Editorial Policies and the Editorial Policy Checklist.

Statistics

For all statistical analyses, confirm that the following items are present in the figure legend, table legend, main text, or Methods section.

n/a	Confirmed	
☐	☒	The exact sample size (*n*) for each experimental group/condition, given as a discrete number and unit of measurement
☐	☒	A statement on whether measurements were taken from distinct samples or whether the same sample was measured repeatedly
☐	☒	The statistical test(s) used AND whether they are one- or two-sided<br>*Only common tests should be described solely by name; describe more complex techniques in the Methods section.*
☐	☒	A description of all covariates tested
☐	☒	A description of any assumptions or corrections, such as tests of normality and adjustment for multiple comparisons
☐	☒	A full description of the statistical parameters including central tendency (e.g. means) or other basic estimates (e.g. regression coefficient) AND variation (e.g. standard deviation) or associated estimates of uncertainty (e.g. confidence intervals)
☐	☒	For null hypothesis testing, the test statistic (e.g. *F*, *t*, *r*) with confidence intervals, effect sizes, degrees of freedom and *P* value noted<br>*Give P values as exact values whenever suitable.*
☐	☒	For Bayesian analysis, information on the choice of priors and Markov chain Monte Carlo settings
☐	☒	For hierarchical and complex designs, identification of the appropriate level for tests and full reporting of outcomes
☐	☒	Estimates of effect sizes (e.g. Cohen's *d*, Pearson's *r*), indicating how they were calculated

Our web collection on statistics for biologists contains articles on many of the points above.

Software and code

Policy information about availability of computer code

Data collection	N/A
Data analysis	Open-source software implementing the ATM method is available at https://github.com/Xilin-Jiang/ATM. BOLT-LMM 2.3 is available at https://alkesgroup.broadinstitute.org/BOLT-LMM/. Heritability and genetic correlation analysis were performed using LDSC, which is available at https://github.com/bulik/ldsc. PLINK v1.9, which was used for FST and association tests, is available at https://www.cog-genomics.org/plink/.

For manuscripts utilizing custom algorithms or software that are central to the research but not yet described in published literature, software must be made available to editors and reviewers. We strongly encourage code deposition in a community repository (e.g. GitHub). See the Nature Portfolio guidelines for submitting code & software for further information.

Data

Policy information about availability of data

All manuscripts must include a data availability statement. This statement should provide the following information, where applicable:

- Accession codes, unique identifiers, or web links for publicly available datasets
- A description of any restrictions on data availability
- For clinical datasets or third party data, please ensure that the statement adheres to our policy

UK Biobank data is publicly available at https://www.ukbiobank.ac.uk/; All of Us data is publicly available at https://allofus.nih.gov; LD-scores and HAPMAP3 SNP list are available at https://data.broadinstitute.org/alkesgroup/LDSCORE.

Human research participants

Policy information about studies involving human research participants and Sex and Gender in Research.

Reporting on sex and gender	All individuals in UK Biobank are included in the analyses, where we used biological sex that are available in UK Biobank.
Population characteristics	N/A
Recruitment	N/A
Ethics oversight	All individuals in UK Biobank are included in the main analyses; for several secondary analyses we constraint our analysis on British Isle ancestry label provided in UK Biobank, which is stated in the text.

Note that full information on the approval of the study protocol must also be provided in the manuscript.

Field-specific reporting

Please select the one below that is the best fit for your research. If you are not sure, read the appropriate sections before making your selection.

☒ Life sciences ☐ Behavioural & social sciences ☐ Ecological, evolutionary & environmental sciences

For a reference copy of the document with all sections, see nature.com/documents/nr-reporting-summary-flat.pdf

Life sciences study design

All studies must disclose on these points even when the disclosure is negative.

Sample size	500,000 UK Biobank participant. No sample size calculation was performed.
Data exclusions	No data were excluded from the main analyses; for several secondary analyses we constraint our analysis on British Isle ancestry label provided in UK Biobank, which is stated in the text.
Replication	All attempts at replication were successful.
Randomization	We used All of Us data to verify the disease topics identified in UK Biobank. We used publicly available data. No participant was recruited.
Blinding	No participant was recruited; the investigators were blinded to group allocation during data collection and/or analysis.

Reporting for specific materials, systems and methods

We require information from authors about some types of materials, experimental systems and methods used in many studies. Here, indicate whether each material, system or method listed is relevant to your study. If you are not sure if a list item applies to your research, read the appropriate section before selecting a response.

Materials & experimental systems

n/a	Involved in the study
☒	☐ Antibodies
☒	☐ Eukaryotic cell lines
☒	☐ Palaeontology and archaeology
☒	☐ Animals and other organisms
☒	☐ Clinical data
☒	☐ Dual use research of concern

Methods

n/a	Involved in the study
☒	☐ ChIP-seq
☒	☐ Flow cytometry
☒	☐ MRI-based neuroimaging
